# The conserved macrodomains of the non-structural proteins of Chikungunya virus and other pathogenic positive strand RNA viruses function as mono-ADP-ribosylhydrolases

**DOI:** 10.1038/srep41746

**Published:** 2017-02-02

**Authors:** Laura Eckei, Sarah Krieg, Mareike Bütepage, Anne Lehmann, Annika Gross, Barbara Lippok, Alexander R. Grimm, Beate M. Kümmerer, Giulia Rossetti, Bernhard Lüscher, Patricia Verheugd

**Affiliations:** 1 Institute of Biochemistry and Molecular Biology, Medical School, RWTH Aachen University, 52057 Aachen, Germany; 2 Institute of Biotechnology, RWTH Aachen University, 52074 Aachen, Germany; 3 Institute of Virology, University of Bonn Medical Centre, 53127 Bonn, Germany; 4 Computational Biomedicine, Institute for Advanced Simulation IAS-5 and Institute of Neuroscience and Medicine INM-9, Forschungszentrum Jülich, 52425, Jülich, Germany; 5 Jülich Supercomputing Centre, Forschungszentrum Jülich, 52425, Jülich, Germany; 6 Department of Oncology, Hematology and Stem Cell Transplantation, Medical School, RWTH Aachen University, Aachen, Germany

## Abstract

Human pathogenic positive single strand RNA ((+)ssRNA) viruses, including Chikungunya virus, pose severe health problems as for many neither efficient vaccines nor therapeutic strategies exist. To interfere with propagation, viral enzymatic activities are considered potential targets. Here we addressed the function of the viral macrodomains, conserved folds of non-structural proteins of many (+)ssRNA viruses. Macrodomains are closely associated with ADP-ribose function and metabolism. ADP-ribosylation is a post-translational modification controlling various cellular processes, including DNA repair, transcription and stress response. We found that the viral macrodomains possess broad hydrolase activity towards mono-ADP-ribosylated substrates of the mono-ADP-ribosyltransferases ARTD7, ARTD8 and ARTD10 (aka PARP15, PARP14 and PARP10, respectively), reverting this post-translational modification both *in vitro* and in cells. In contrast, the viral macrodomains possess only weak activity towards poly-ADP-ribose chains synthesized by ARTD1 (aka PARP1). Unlike poly-ADP-ribosylglycohydrolase, which hydrolyzes poly-ADP-ribose chains to individual ADP-ribose units but cannot cleave the amino acid side chain - ADP-ribose bond, the different viral macrodomains release poly-ADP-ribose chains with distinct efficiency. Mutational and structural analyses identified key amino acids for hydrolase activity of the Chikungunya viral macrodomain. Moreover, ARTD8 and ARTD10 are induced by innate immune mechanisms, suggesting that the control of mono-ADP-ribosylation is part of a host-pathogen conflict.

ADP-ribosylation describes a posttranslational modification (PTM) in which an ADP-ribose (ADPr) moiety is transferred from NAD^+^ onto substrate proteins with release of nicotinamide. Intracellular ADP-ribosylation is mainly catalyzed by enzymes of the ADP-ribosyltransferase diphtheria toxin-like (ARTD) family (also known as PARP family)^1^. Based on their biochemical features ARTDs can be subdivided into three groups: members of the first group (including ARTD1/2/5/6) are able to iteratively transfer multiple ADPr units onto their substrates resulting in the formation of long branched ADPr polymers (PAR). Group II enzymes (ARTD7/8/10-12/14-17) are restricted to mono-ADP-ribosylation (MARylation), in part due to the lack of a catalytically active glutamate. The latter have been suggested to use substrate-assisted catalysis to modify their targets^2^,^3^. Whether ARTD3 and 4 belong to group I or II, despite both having a catalytic glutamate, is somewhat controversial and requires further analyses^3^. Acceptor amino acids for ADP-ribosylation are still a matter of debate with some discrepancy between biochemical and mass spectrometry studies. Nevertheless, acidic amino acids are considered important acceptor sites for both group I and II enzymes^4^. Our own findings with ARTD10 strongly suggest that glutamates are the main sites of modification^2^. Proteins of the third group (ARTD9/13) lost the ability to bind NAD^+^ due to amino acid substitutions in the NAD^+^ binding pocket and therefore are catalytically inactive^1^,^5^. Recent evidence defines reader domains that are capable of interacting specifically with MARylated or poly-ADP-ribosylated (PARylated) substrates and thus participate in disseminating the information associated with this PTM^6^,^7^. Moreover, erasers have been identified that hydrolyze bonds between single ADPr units and between ADPr and the modified amino acid, defining ADP-ribosylation as a fully reversible PTM^7^,^8^.

A key protein fold involved in both reading and erasing ADP-ribosylation is the macrodomain, an evolutionary conserved structural domain^3^,^7^,^8^,^9^. Several macrodomains, including the one of the core histone macroH2A1.1, interact with ADPr polymers^7^,^9^. Others bind selectively to MARylated substrates, as shown for macrodomain 2 (macro2) or macrodomain 3 (macro3) of murine Artd8^10^. The macrodomains of ARTD7 are poorly characterized, while those of ARTD9 have been suggested to affect transcription and the DNA damage response^7^,^8^. Importantly, some macrodomains possess enzymatic activity. For example, the macrodomain of poly-ADP-ribosylglycohydrolase (PARG) degrades PAR chains, whereas the macrodomains of TARG1, MacroD1 and MacroD2 remove the terminal, protein bound ADPr unit^11^,^12^,^13^,^14^. Thus the latter enzymes hydrolyze the ester bond between the ADPr and a most likely acidic acceptor amino acid^11^,^12^. Together these findings document the important role of macrodomain folds in regulating ADP-ribosylation function and metabolism.

ADP-ribosylation is implicated in a variety of biological processes including DNA repair, chromatin remodeling, mitosis, transcription, and signaling^4^,^6^,^15^. A growing body of information suggests functions for MARylation at the interface between host and pathogens^3^. On the one hand MARylation of host proteins is catalyzed by a range of bacterial toxins, thereby promoting pathogenesis^16^. Thus MARylation is a conserved mechanism for host protein modulation to promote virulence. On the other hand accumulating evidence links intracellular MARylation to the innate immune response^17^. Infection of human monocytes by *Borrelia burghdorferi* results in elevated expression of *ARTD8, 10* and *12*^18^. In line with these findings, the expression of these three genes is also induced by interferon (IFN) responsive factor 1 as part of an antiviral program^19^. Moreover, monitoring the IFN response by a reporter gene construct defined *ARTD10* as a type I IFN-stimulated gene^20^. *ARTD7, 10* and *12* are induced upon IFNβ stimulation and inhibit alphavirus replication^21^. Furthermore, these proteins participate in repression of protein translation when combined with viral infection^21^,^22^. In addition to these molecular and cell biological studies, evolutionary analyses indicate a broad role of ADP-ribosylation in virus-host interactions^23^. Several ARTD family members are under strong recurrent positive selection, including *ARTD7, 8* and *9*, the three ARTD family members possessing macrodomains as well as the catalytically inactive *ARTD13*^23^. The latter has been well established in antiviral immune defense. ARTD13 interferes with viral RNA translation and replication by interaction with viral RNA through its four CCCH-type zinc fingers thereby promoting its degradation^3^. Taken together these findings strongly imply important functions for ARTD family members in innate immunity and thus suggest that ADP-ribosylation and its functional consequences contribute to the resolution of host-pathogen conflicts.

As mentioned above, macrodomains can function as readers as well as erasers of MARylation^7^,^8^. The fact that macrodomains are also conserved domains of non-structural proteins of some positive single strand RNA ((+)ssRNA) viruses provides further evidence for a function of ADP-ribosylation in innate immunity^24^,^25^. These ((+)ssRNA) viruses include members of the alphavirus genus such as Chikungunya virus (CHIKV), O’nyong’nyong virus (ONNV), Sindbis virus (SINV), and Venezuelan Equine Encephalitis virus (VEEV); members of the orthohepevirus genus among them Hepatitis E virus (HEV), as well as members of the alphacoronavirus genus, to which Feline Infectious Peritonitis virus (FIPV), a mutant of Feline coronavirus, belongs. Some of these viruses are human pathogens. For example CHIKV, transmitted by mosquitoes, has resulted in large epidemics in recent years. One of its effects is severe joint pain that can last for months or even years, thus having considerable long-term effects on human health^26^,^27^. CHIKV, among other (+)ssRNA viruses, is one of the top rated pathogens that is in need for vaccine development^28^.

The viral macrodomains (vMDs) of the above mentioned viruses are only poorly characterized with regard to biochemical properties and biological functions. Some of the vMDs were shown to bind free ADPr as well as PAR^25^, while ADP-ribose-1″-monophosphatase (ADRP) activity was assigned to others^24^,^29^,^30^. Moreover, severe acute respiratory syndrome (SARS) coronavirus harbors two additional macrodomain folds in the SARS-unique domain, which lack ADPr binding, but instead interact with G-quadruplexes^31^,^32^,^33^,^34^. Very recently ADP-ribosylhydrolase activity has been reported for the viral macrodomains of HEV, SARS (the macrodomain fold of the X domain), and VEEV^35^. Several findings provide evidence for a biological relevance of vMDs. Mutation of the ADPr binding region of the SINV macrodomain of the non-structural protein 3 (nsP3) impairs viral replication in neurons^36^. Mutations in the vMD with ADRP activity of SARS coronavirus and human coronavirus 229E resulted in increased sensitivity to the antiviral effect of IFNα compared with their wildtype counterparts^37^. Moreover, the mouse hepatitis virus macrodomain promotes virulence in mice^38^. vMDs thus seem to be critical for viral pathogenesis and evasion of the host immune response.

Here we have further evaluated the interplay of ADP-ribosylation and vMDs. We demonstrate that the genes encoding ARTD8 and ARTD10 are targets of IFNα signaling and thus are likely components of a first line immune response to viruses. Furthermore, we provide a detailed biochemical analysis of diverse vMDs revealing their hydrolase activity towards MARylated proteins. In contrast, PAR chains are only poorly targeted by the tested vMDs. We focused on the CHIKV macrodomain. By using bioinformatics approaches combined with the information obtained from the crystal structures of vMDs in complex with ADPr^25^,^39^, we successfully generated mutants, which were unable to de-MARylate substrates. Complementary to our *in vitro* analyses, the CHIKV macrodomain reverts MARylation in cells. This novel enzymatic activity of vMDs strongly underlines the hypothesis that MARylation functions at the host-pathogen interface and thus is a potential drug target.

## Results

### Mono-ARTDs are regulated by interferon

**Figure 1:**
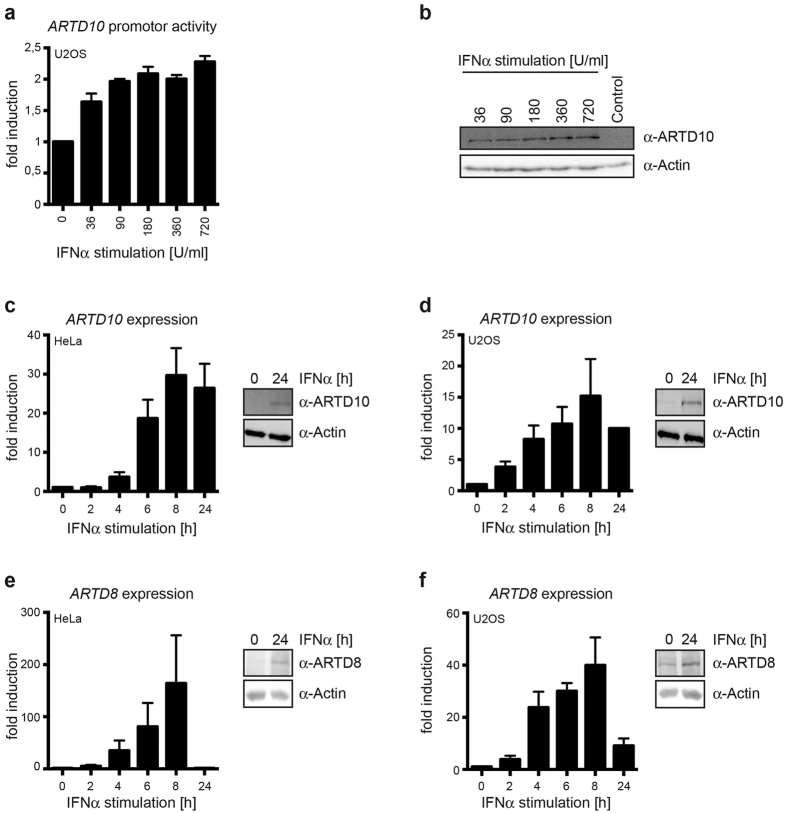
Expression of ARTD8 and ARTD10 is stimulated by interferon α. (**a**) A fragment of the human *ARTD10* promoter (−532 to +491 relative to the start of transcription) was cloned in front of a luciferase reporter gene. This construct was transfected together with a β-galactosidase expressing plasmid into U2OS cells, which were then stimulated with the indicated amounts of interferon (IFN) α. 24 h later the relative luciferase activity was determined. Mean values ± SD of three experiments. (**b**) Untransfected U2OS cells were treated with IFNα in parallel to the cells in panel A and whole cell lysates analyzed for ARTD10 expression by immunoblotting using mAb 5H11. (**c**–**f**) HeLa and U2OS cells were stimulated with IFNα for the indicated times. The expression of *ARTD10* and *ARTD8* mRNA was measured using RT-qPCR (mean values ± SD of three experiments). ARTD10 and ARTD8 protein was analyzed by immunoblotting using mAb 5H11 (panels c and d) and peptide-specific pAb (panels e and f), respectively.

In order to identify stimuli driving *ARTD10* expression, a fragment of the *ARTD10* promoter (−532 to +491 relative to the start of transcription) was cloned into the pGL3 basic luciferase reporter gene construct. U2OS cells were transiently transfected with this reporter gene construct and stimulated with the antiviral defense-inducing cytokine IFNα. This stimulated the *ARTD10* promoter reporter gene construct in a dose dependent manner (Fig. 1a), in accordance with previous findings^20^. IFNα also promoted endogenous ARTD10 protein synthesis in these cells (Fig. 1b). Next we analyzed endogenous *ARTD10* gene expression. IFNα stimulated *ARTD10* mRNA and protein synthesis in HeLa and U2OS cells (Fig. 1c and d), consistent with our reporter gene assays. Similarly to the findings for *ARTD10, ARTD8* expression was also induced by IFNα resulting in elevated mRNA and protein levels in HeLa and U2OS cells (Fig. 1e and f). To detect ARTD8 protein levels we made use of an antibody generated against a peptide derived from the sequence located between macrodomain 2 and 3 of ARTD8 (Supplementary Fig. S1a). Analogous to IFNα, lipopolysaccharide (LPS), a bacterial pathogen-associated molecular pattern, stimulated *ARTD10* mRNA and protein synthesis in differentiated THP-1 cells (Supplementary Fig. S1b), but not in undifferentiated cells (data not shown). Of note is that both IFNα and LPS stimulated *ARTD10* and *ARTD8* expression with some delay, indicating that the induction of these genes is probably indirect rather than through direct activation of IFN receptor downstream signaling events. Taken together our results support the notion that mono-ARTDs like ARTD8 and ARTD10 play a role in the first line immune response that is induced in consequence of stimuli such as IFNα and LPS. Our observations and published findings let us hypothesize a function for MARylation in the immune defense against pathogens.

### Viral macrodomains possess hydrolase activity towards mono-ADP-ribosylated substrates

**Figure 2:**
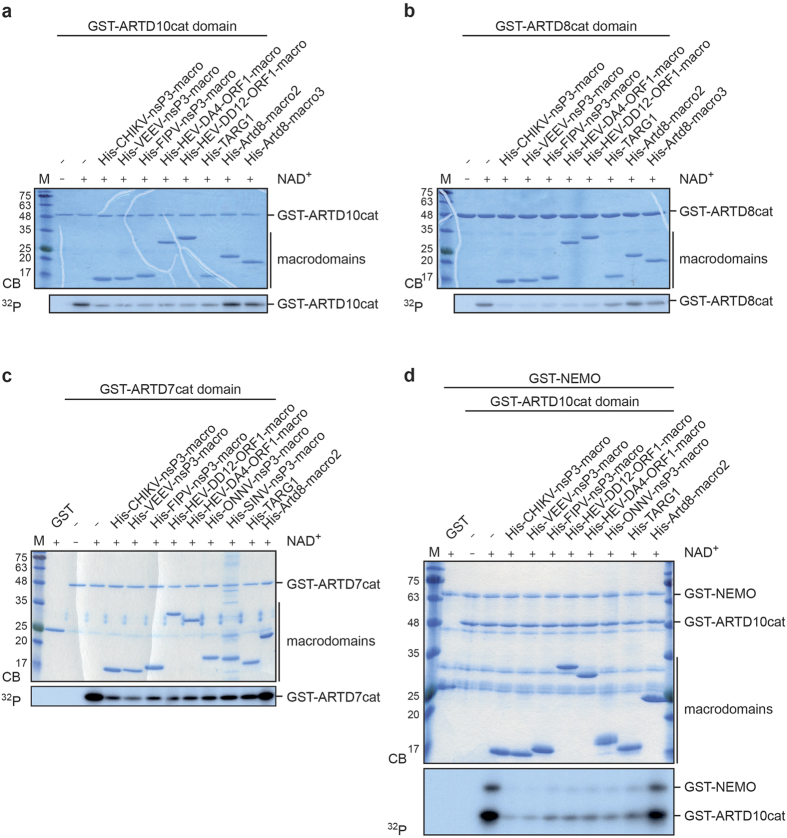
Viral macrodomains possess mono-ADP-ribosylhydrolase activity. (**a**–**d**) Bacterially expressed and purified ARTD catalytic domains (ARTD10 in panels a and d, ARTD8 in panel b, and ARTD7 in panel c) were automodified in the presence of ^32^P-NAD^+^. In addition, the ARTD10 substrate NEMO was included (panel d). The modified proteins were then incubated with the indicated bacterially expressed and His_6_-tagged fusion proteins of different macrodomains for 60 min. The proteins were stained using Coomassie blue (CB) and the radioactivity associated with the different substrates was assessed by autoradiography (^32^P). CHIKV, Chikungunya virus; VEEV, Venezuelan Equine Encephalitis virus; FIPV, Feline Infectious Peritonitis virus; HEV, hepatitis E viruses (strains XDD12 and XDA4); ONNV, O’nyong’nyong virus; SINV, Sindbis virus; Artd8-macro2, macrodomain 2 of murine Artd8; Artd8-macro3, macrodomain 3 of murine Artd8; nsP3, non-structural protein 3; ORF1, open reading frame 1 protein; CB, Coomassie blue; ^32^P, autoradiogram.

**Figure 3:**
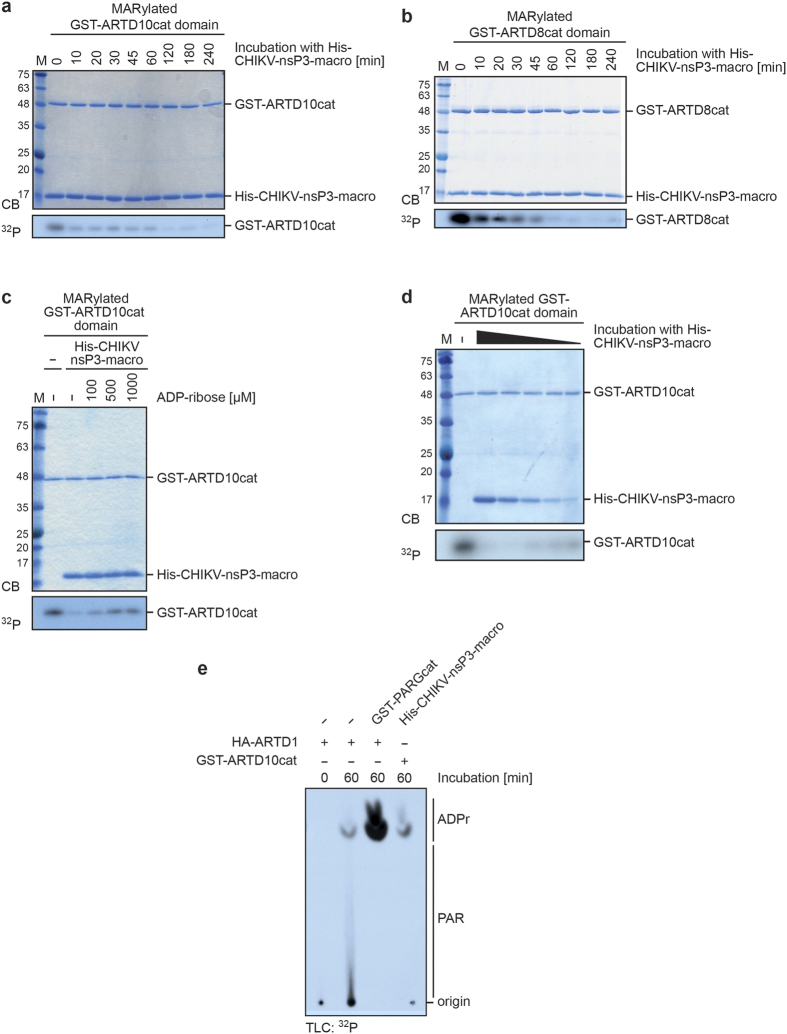
Viral macrodomains are specific and efficient hydrolases. (**a**) GST-ARTD10cat domain was automodified in the presence of ^32^P-NAD^+^. The proteins were then incubated with CHIKV-nsP3 macrodomain for the indicated times. The proteins were stained using Coomassie blue (CB) and the radioactivity associated with the different substrates was assessed by autoradiography (^32^P). (**b**) As in panel a but with the automodified catalytic domain of ARTD8 as substrate. (**c**) As in panel a but in presence of increasing amounts of ADP-ribose. (**d**) As in panel a but the CHIKV-nsP3-macro was titrated (1:2 serial dilutions). (**e**) Immunoprecipitated HA-ARTD1 or GST-ARTD10cat domain were automodified in presence of ^32^P-NAD^+^. The proteins were then incubated with PARGcat (ARTD1) or CHIKV-nsP3 macrodomain (ARTD10) at 30 °C for the indicated times. The released, radioactively labeled products were analyzed by subjecting the supernatants of the reactions to thin layer chromatography (TLC). CB, Coomassie blue; TLC, thin layer chromatography; ^32^P, autoradiogram.

To expand on this hypothesis, we were interested in functionally characterizing vMDs. As outlined above certain macrodomains are linked to MARylation either by interacting with MARylated substrates or by removing this modification. Therefore, we assessed whether the tested vMDs, besides binding to free ADPr, are able to remove MARylation from substrates by hydrolyzing the bond between amino acid side chains and the ribose, analogously to the three cellular mono-ADP-ribosylhydrolases TARG1, MacroD1 and MacroD2^11^,^12^,^14^. We purified His_6_-tagged fusion proteins of several vMDs, TARG1, Artd8-macro2 and -macro3 and performed hydrolase assays on automodified, MARylated GST-fusion proteins of the catalytic domains of ARTD10, ARTD8, and ARTD7, and on MARylated NEMO, an ARTD10 substrate^40^ (Figs 2 and 3, Supplementary Fig. S2). The proteins were MARylated using ^32^P-NAD^+^ and subsequently incubated with the indicated macrodomain fusion proteins. We observed that all vMDs tested, i.e. those of CHIKV, VEEV, FIPV, SINV, ONNV and HEV, which are closely related (Supplementary Fig. S3), were capable of removing a substantial fraction of the ^32^P-labeled MARylation from ARTD7, ARTD8, ARTD10, and NEMO after 60 min. As expected, TARG1 was also catalytically active, whereas macro2 and macro3 of Artd8 were unable to hydrolyze MARylation^10^,^14^ (Fig. 2). These findings suggest that the vMDs possess hydrolase (de-MARylation) activity towards several MARylated substrates, including NEMO, a component of the NF-κB signal transduction pathway^40^.

To further characterize the enzymatic reaction, we performed time-course experiments with His_6_-tagged vMDs of CHIKV, FIPV, ONNV, and SINV on MARylated catalytic domains of GST-ARTD10 and GST-ARTD8 (Fig. 3a and b; Supplementary Fig. S2). Up to roughly half of the incorporated ^32^P label was released by the vMDs within 10 min and most of the label after 120 to 240 min, with small differences between individual vMDs.

Based on the known interaction of vMDs with ADPr, we expected ADPr to be a competitive inhibitor of vMDs. Indeed, free ADPr inhibited hydrolase activity of the CHIKV macrodomain towards the MARylated ARDT10 catalytic domain (Fig. 3c), similar to findings observed for MacroD2^11^,^25^. The inhibitory effect of ADPr was concentration dependent. Titration of the CHIKV macrodomain further demonstrated robust hydrolase activity. Even at low protein concentrations the CHIKV macrodomain reversed MARylation efficiently (Fig. 3d). The products released by the CHIKV macrodomain were analyzed by thin layer chromatography (TLC) and found to migrate identically to the products obtained from PARGcat-treated, automodified ARTD1 (Fig. 3e). PARG hydrolyses efficiently the ribose-ribosyl glycosidic bonds between ADPr units in PAR chains, generating ADPr^41^. Thus these findings are consistent with release of ADPr by the CHIKV macrodomain. Together these results define vMDs as ADP-ribosylhydrolases capable of efficiently reverting mono-ADP-ribosylation catalyzed by three different mono-ARTDs.

### Poly-ADP-ribose chains are inefficiently hydrolyzed by viral macrodomains

**Figure 4:**
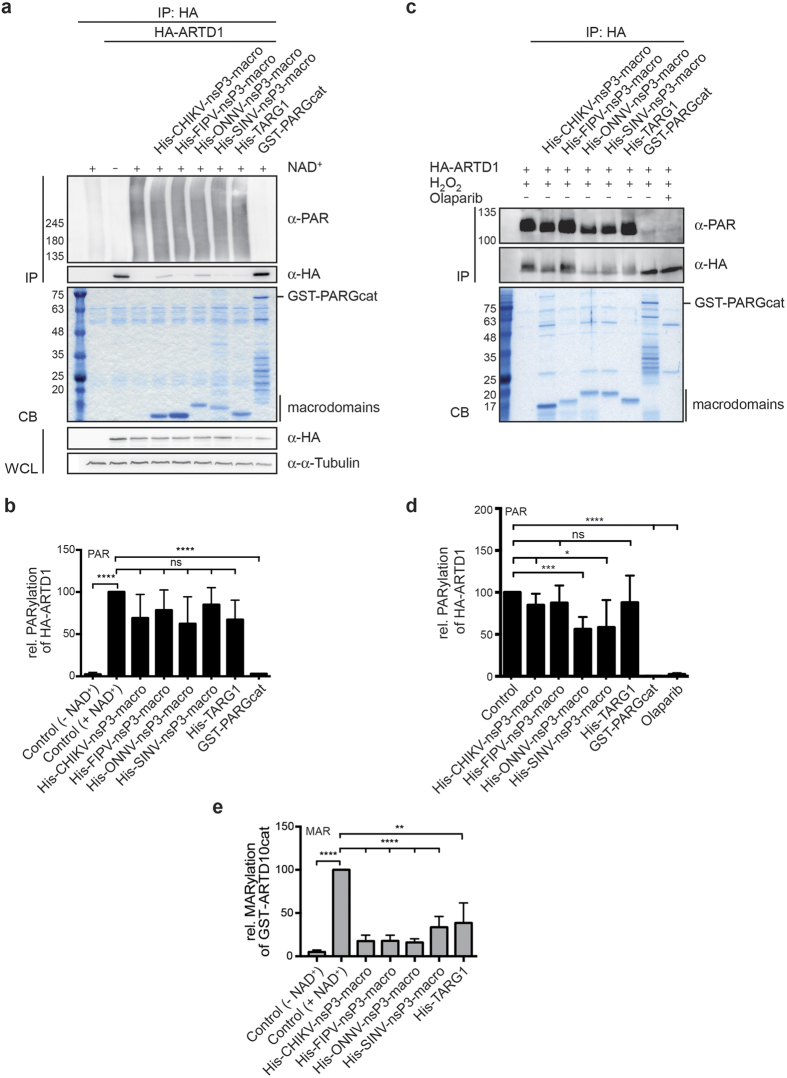
Poly-ADP-ribose chains are removed inefficiently by viral macrodomains. (**a**) HA-ARTD1 was expressed in HEK293 cells, immunoprecipitated from cell lysates and automodified by incubation with NAD^+^ in the presence of double stranded oligomers. The PARylated ARTD1 was then incubated with the indicated macrodomains at 30 °C for 60 min. PAR chains were evaluated using a PAR-specific antibody. The proteins were stained using Coomassie blue (CB). For control the expression of HA-ARTD1 and of α-Tubulin was determined in whole cell lysates (WCL) by immunoblotting. (**b**) Experiments were performed as in panel a and quantified. Mean values ± SD of 3 experiments are shown. (**c**) HeLa cells, which transiently express HA-ARTD1, were treated with H_2_O_2_ for 7 min. Immunoprecipitated HA-ARTD1 was then treated with the indicated macrodomains for 60 min and PARylation evaluated using a PAR-specific antibody on Immunoblots. For control HA-ARTD1 was incubated with PARGcat domain or isolated from Olaparib-treated cells. (**d**) Experiments were performed as in panel c and quantified. Mean values ± SD of 3 experiments are shown. (**e**) Quantification of ARTD10 de-MARylation measured at 60 min. Mean values ± SD of 3–5 experiments are shown. CB, Coomassie blue; IP, immunoprecipitation; WCL, whole cell lysate. ****p < 0.0001; ***p < 0.001; **p < 0.01; ns, not significant when an unpaired *t*-test was applied.

To determine whether vMDs, in addition to removing mono-ADPr from substrates, also possess activity towards PAR chains, we performed hydrolase assays on ARTD1 that was PARylated *in vitro* or in cells (Fig. 4, Supplementary Figs S4 and S5). HA-tagged ARTD1 was transiently expressed in HEK293 cells. Cells were harvested and ARTD1 was immunoprecipitated using HA-specific antibodies from whole cell lysates. Auto-PARylation of immunoprecipitated ARTD1 was stimulated by the addition of double stranded DNA fragments in the presence of β-NAD^+^. Subsequently hydrolase assays were performed and PARylation was measured using a PAR-specific antibody (Fig. 4a). Quantification of the activities of the vMDs of CHIKV, FIPV, ONNV, and SINV showed weak activity towards *in vitro* PARylated ARTD1, nevertheless, we observed a trend to some reduction in PARylation (Fig. 4b). Moreover, an increase in the signal was measured with the HA-specific antibodies in response to the vMDs of CHIKV and ONNV suggesting that some of the ARTD1 molecules were no longer shifted to higher mobility, indicative of some activity against modified ARTD1 (Fig. 4a, see α-HA of the immunopreciptiated (IP) material). Similarly, no significant activity of TARG1 was measurable in these assays. In contrast, incubation with the catalytic domain of PARG, a well-described eraser of PARylation with a macrodomain fold^13^, resulted in a nearly complete loss of the PAR signal and an α-HA staining of the immunopreciptiated HA-ARTD1 comparable to the negative control that was incubated in the absence of β-NAD^+^ (Fig. 4a and b).

These findings were corroborated by analyzing immunoprecipitated ARTD1, which was isolated from transiently transfected HeLa cells that were treated with H_2_O_2_ prior to harvesting to stimulate intracellular PARylation (Fig. 4c). The immunoblot analyses indicated that the modifications obtained in cells were less substantial, i.e. shorter PAR chains, compared to ARTD1 modified *in vitro* (compare panels a and c of Fig. 4). Treatment of the cells with the ARTD1 inhibitor Olaparib as well as incubation with PARGcat served as control^42^,^43^. Moreover, Olaparib was added to the lysis buffer to prevent automodification during cell lysis. In agreement with the results from the *in vitro* modified ARTD1 above, PARylation was affected to some extend dependent on the identity of the vMD (Fig. 4c and d). But in contrast to the *in vitro* assays, the reductions in response to the CHIKV, ONNV and SINV macrodomains were significant, although not comparable to the effects in response to incubation with PARGcat (Fig. 4d). For direct comparison we have quantified MAR levels on ARTD10cat incubated with the vMDs after 60 min of hydrolysis (Fig. 4e). The results suggest that all vMDs tested are considerably more efficient in de-MARylating ARTD10 compared to de-PARylating ARTD1.

The findings summarized in Fig. 4 were obtained by incubating the samples for only 60 min. But the time course experiments with different vMDs on MARylated substrates demonstrated that the de-MARylation further increased over longer time periods up to a very substantial, almost complete loss of the modification after 240 min (Fig. 3 and Supplementary Fig. S2). Therefore, we assessed de-PARylation of *in vitro* modified HA-ARTD1 by the macrodomains of CHIKV and FIPV over time (Supplementary Fig. S4). The CHIKV but not the FIPV macrodomain exhibited weak hydrolase activity when compared to the control incubated for 240 min in the absence of a vMD. It is of note that the latter incubation of ARTD1 for 240 min on its own already exhibited a substantial reduction of PARylation when compared to the starting point and some de-PARylation without added macrodomains was also seen by others^35^. These findings are in agreement with the analysis of the released products of automodified HA-ARTD1 (Supplementary Fig. S5). Incubation in the absence of any macrodomain resulted in the release of some ADPr as well as PAR chains already after 60 min, as measured by TLC analysis. This was slightly stimulated by the different vMDs, with the exception of the macrodomain of FIPV (Supplementary Fig. S5a). Again, TARG1 showed no activity and PARG resulted in the complete hydrolysis to ADPr. These results were corroborated by analyzing the released ^32^P-labeled PAR chains on sequencing gels and the remaining protein-bound PAR by SDS-PAGE (Supplementary Fig. S5b and c). Based on these findings we suggest that MARylated proteins are the preferred substrates of the different vMDs analyzed, but apparently at least some of the vMDs can gain access to the amino acid side chain - ribose bond of a PAR modified substrate and thus remove complete PAR chains. We have not observed that vMDs can hydrolyze the ribose-ribosyl glycosidic bonds within PAR chains in contrast to PARG.

### Mutational analysis of the CHIKV macrodomain defines catalytically important amino acids

**Figure 5:**
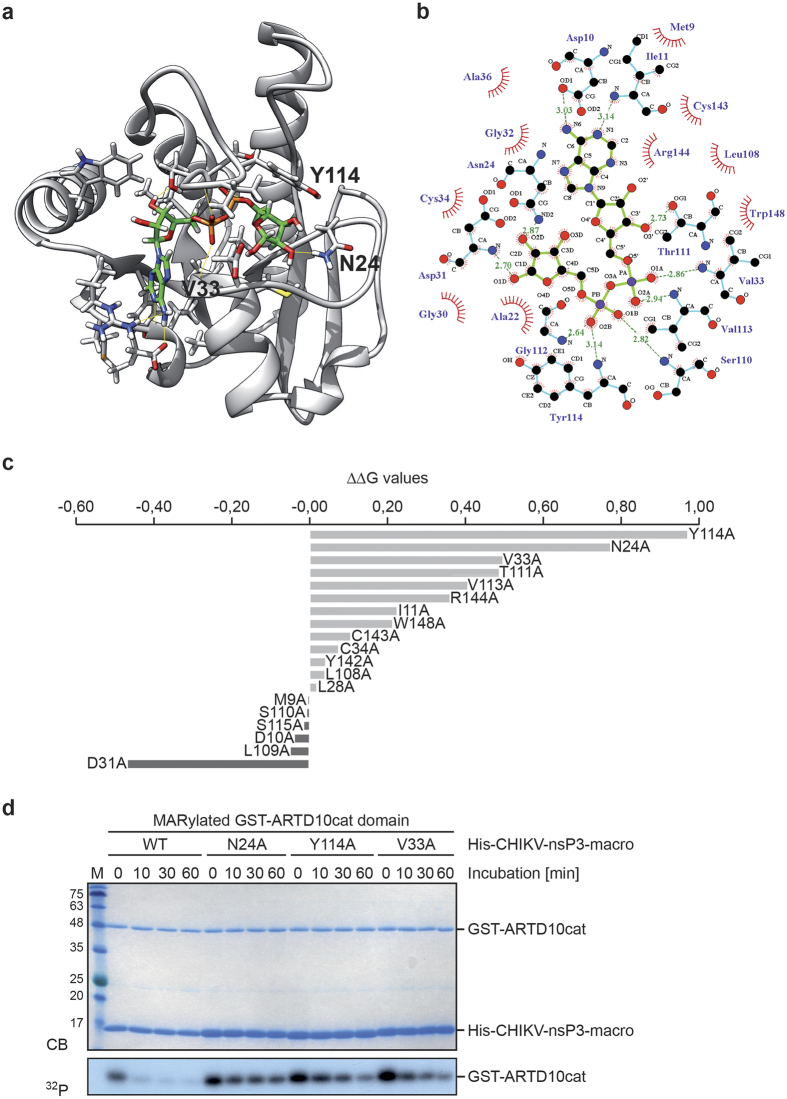
Identification of amino acids in the CHIKV macrodomain that are important for catalysis. (**a**) Structural model of the CHIKV macrodomain derived from the crystal structure (PDBID 3GPO). Highlighted are the amino acids that are relevant for ADPr binding and thus important for catalytic activity. The protein is in grey cartoon representation. ADPr and the binding site residues involved in ligand coordination are depicted in green and grey licorice, respectively. Hydrogen bonds are depicted as yellow lines. (**b**) 2D representation of ADPr in complex with the CHIKV macrodomain is shown based on the LIGPLOT program. ADPr is shown in green lines. Hydrophobic contacts are represented by an arc with spokes radiating towards the ligand atoms they contact. The contacted atoms are shown with spokes radiating back. The carbon atoms, oxygen atoms, and nitrogen atoms are colored in black, red and blue, respectively. The hydrogen bonds that are defined by the crystal structure (panel a) of CHIKV-nsP3-macro with ADPr are depicted in green dashed lines. (**c**) *In silico* alanine scan and ΔΔG values for ADPr binding. Mutation of Y114, N24, and V33 to alanine showed the highest potential effect on ADPr binding. (**d**) CHIKV macrodomain mutants with N24A, V33A and Y114A were expressed as His_6_-tagged fusion proteins in bacteria. Then the purified proteins were incubated with automodified GST-ARTD10cat domain for the indicated times. The proteins were visualized by Coomassie blue staining (CB) and de-MARylation activity of CHIKV macrodomain mutants was determined by autoradiography (^32^P). CB, Coomassie blue; ^32^P, autoradiography.

**Figure 6:**
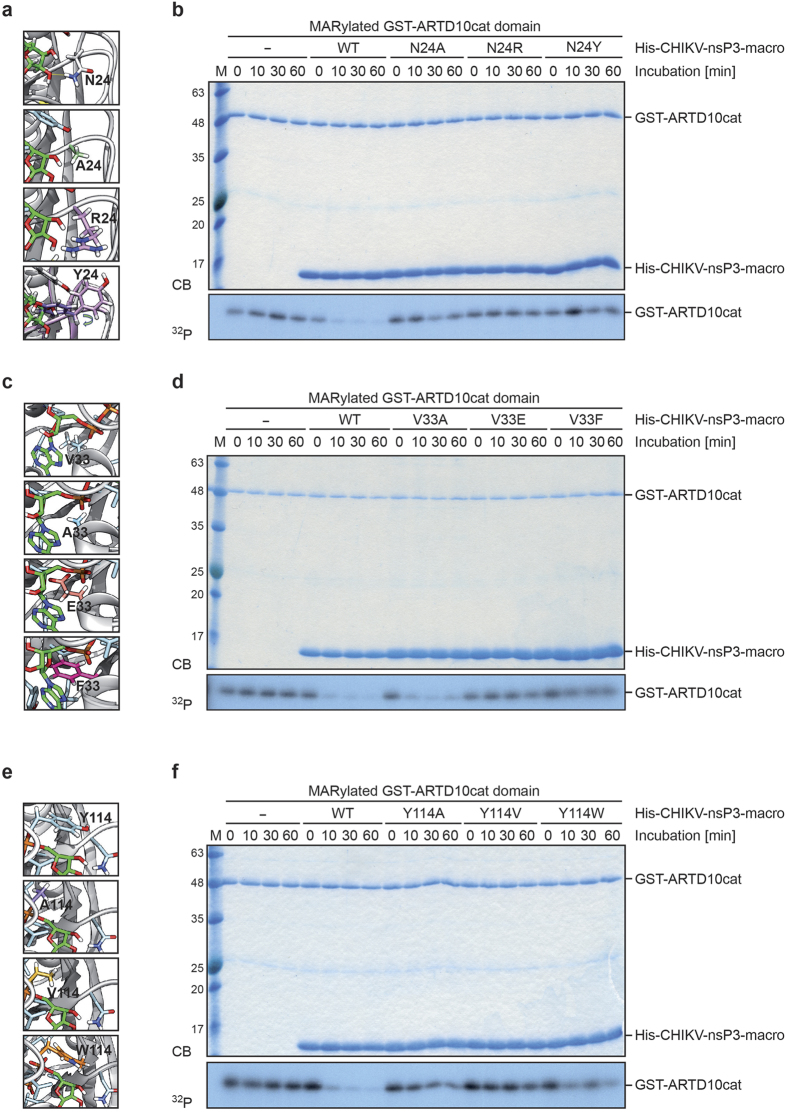
Identification of CHIKV macrodomain mutants that are catalytically inactive. (**a**) Focus on amino acid N24 of the structural model of the CHIKV macrodomain. The models of the mutants obtained by substitutions of position 24 with alanine, arginine and tyrosine are shown. The protein is in grey cartoon representation, while the ADPr is in green licorice representation. (**b**) CHIKV macrodomain mutants with N24R and N24Y, both having a higher Amber score than N24A, were expressed as His_6_-tagged fusion proteins in bacteria. Mutant proteins were evaluated by incubation with automodified GST-ARTD10cat domain for the indicated times. Proteins were visualized by Coomassie blue (CB) staining and the activities of the CHIKV macrodomain mutants were determined by autoradiography (^32^P). (**c**) Focus on amino acid V33 of the structural model of the CHIKV macrodomain. The models of the mutants obtained by substitutions of position 33 with alanine, glutamate and phenylalanine are shown. Coloring is as in panel a. (**d**) CHIKV macrodomain mutants with V33E and V33F, both having a higher Amber score than V33A, were expressed as His_6_-tagged fusion proteins in bacteria. Mutants were analyzed as described in panel b. (**e**) Focus on amino acid Y114 of the structural model of the CHIKV macrodomain. The models of the mutants obtained by substitution of position 114 with alanine, valine and tryptophan. Coloring is as in panel a. (**f**) CHIKV macrodomain mutants with Y114V and Y114W, both having a higher Amber score than Y114A, were expressed as His_6_-tagged fusion proteins in bacteria. Mutants were analyzed as described in panel b. CB, Coomassie blue; ^32^P, autoradiogram.

The catalytic mechanism of macrodomain ADP-ribosylhydrolases is not fully understood^11^,^12^,^13^. To obtain information about amino acids relevant for catalytic activity, we focused on the published crystal structure of the CHIKV macrodomain in complex with ADPr^25^. This was chosen as no structural information of a macrodomain in complex with a MARylated substrate, i.e. peptide or protein, has been reported thus far. We performed *in silico* analyses to identify amino acids facing the ADPr binding pocket that contribute to ADPr binding (Fig. 5a and b). The procedure allowed identifying important amino acids for ligand recognition. All amino acids within a distance of 5 Å from the ADPr were subjected to *in silico* alanine (A) scanning. The obtained mutants were ranked according to the difference in interaction score with respect to the wildtype (WT) protein (∆∆G values) (Fig. 5c). For each of the three best hits (arginine (N) 24, valine (V) 33, and tyrosine (Y) 114) site-directed mutagenesis of the WT CHIKV macrodomain was performed and the amino acids were changed to alanine (N24A, V33A, and Y114A). His_6_-tagged fusion proteins of the CHIKV macrodomain mutants were purified and subjected to hydrolase assays of MARylated GST-ARTD10cat for the indicated times (Fig. 5d). The vMD mutants showed less hydrolase activity compared to the WT protein, indicating that the three identified amino acids are important for substrate binding and/or catalysis, but still exhibited a visible de-MARylation. To obtain macrodomain mutants with a more substantial loss of enzymatic activity, we evaluated the energetics of ligand/protein interaction for every possible amino acid substitution of each of the three positions (Supplementary Table S1). Based on these scores we generated additional mutants (Fig. 6). We replaced N24 with arginine (R) and tyrosine (Y) (Fig. 6a), V33 with glutamate (E) and phenylalanine (F) (Fig. 6c), and Y114 with valine (V) and tryptophan (W) (Fig. 6e). In subsequent hydrolase assays with MARylated ARTD10cat as substrate all mutants showed reduced catalytic activity, with N24R, N24Y, V33E, and Y114V being inactive compared to the control incubations in the absence of vMD protein (Fig. 6b, d and f). In addition to ARTD10, we evaluated the hydrolase activity of the V33E and Y144V mutant towards PARylation catalyzed by ARTD1. Therefore, hydrolase assays were performed as described before and the released products were analyzed by TLC (Supplementary Fig. S6a). The mutants were inactive, analogous to the results obtained with the MARylated ARTD10cat domain as substrate. N24 faces the ADPr binding pocket and forms a hydrogen bond with the substrate-linked ribose ring (Fig. 5b). In this position it might also influence the recognition of the substrate, i.e. a MARylated protein. When mutated to A, R or Y this hydrogen bond is lost. In addition, the N24Y mutant, due to the prediction that the tyrosine can rotate (Fig. 6a, arrow), might have reduced substrate binding. Moreover, the N24R is suggested to affect the positioning of the ligand and as a consequence may also reduce substrate binding. V33 is deeply buried in the binding cavity and forms a hydrogen bond between its backbone nitrogen and the diphosphate of ADPr (Figs 5b and 6c). This hydrogen bond can be maintained in the V33A mutant. However, when bulky amino acids like E or F replace V33, they might reduce the accessible volume of the binding site, and therefore hamper the correct binding of the substrate. Moreover, specifically for the V33E mutant, the negative charge of E might also introduce an electrostatic repulsion effect with the phosphate groups of the ADPr. Although not obvious from the crystal structure but based on molecular dynamics^39^, hydrogen bonds are formed between Y114 and the diphosphate as well as the terminal ribose ring of ADPr. Similar to N24 it might also play a role in substrate recognition. When substituted with A, V or W the hydrogen bond with the terminal ribose ring is lost. Taking together N24, V33, and Y114 are important for ligand binding and possibly substrate recognition (N24 and Y114) and thus are necessary for enzymatic activity of the CHIKV macrodomain.

To obtain more information about the consequences of these mutations with regard to structural integrity of the macrodomain fold, the purified CHIKV macrodomain proteins were analyzed using circular dichroism (CD) spectroscopy. All mutants showed a spectrum very similar to the WT protein, indicating that the overall structure was not or only minimally affected by the different point mutations (Supplementary Fig. S6b and c). Thus, our findings suggest that the catalytically inactive mutants are structurally intact but have impaired ADPr and possibly altered substrate binding, providing a molecular explanation for the biochemical observations.

### CHIKV-nsP3 full-length possesses hydrolase activity similar to the isolated vMD

**Figure 7:**
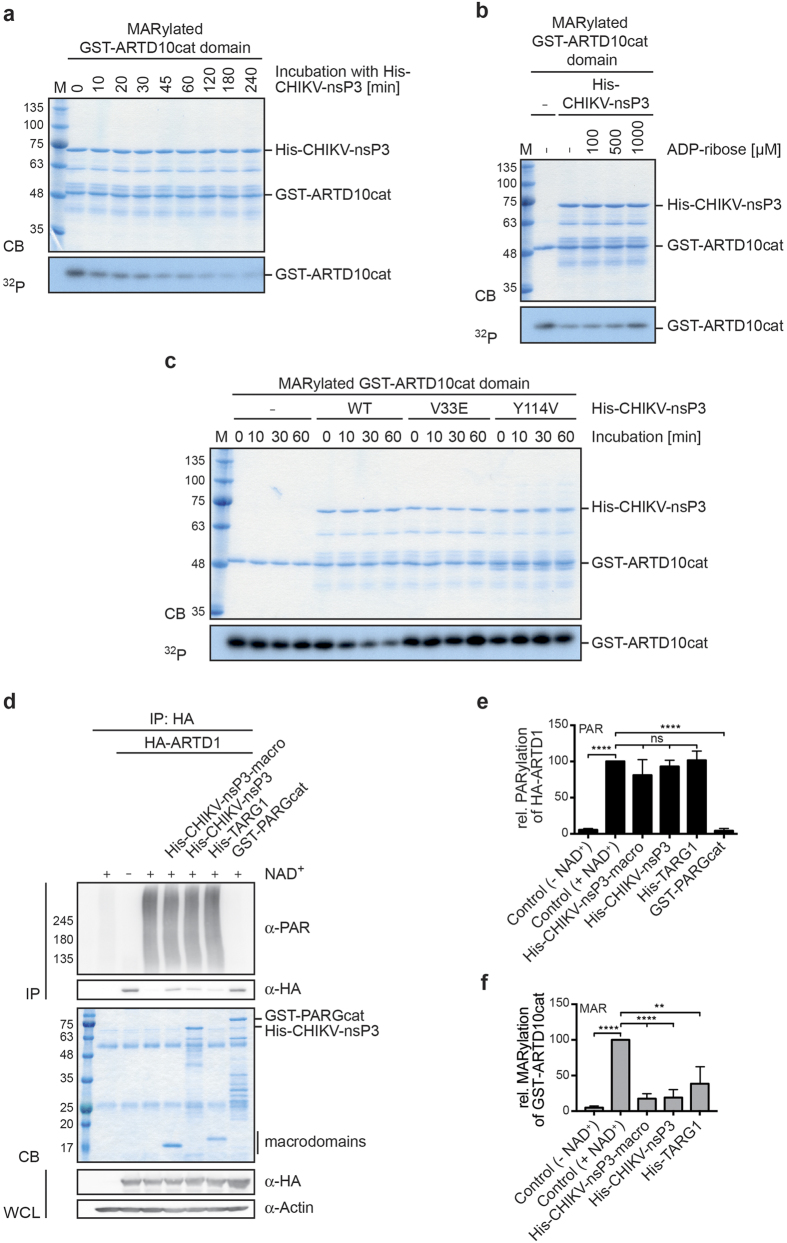
The full length nsP3 protein of CHIKV shows ADP-ribosylhydrolase activity. (**a**) GST-ARTD10cat domain was automodified in the presence of ^32^P-NAD^+^. The proteins were then incubated with CHIKV-nsP3 full length (with the N-terminally located viral macrodomain) for the indicated times. The proteins were visualized by Coomassie blue staining (CB) and catalytic activity of nsP3 was determined by detecting the incorporated radioactive label by autoradiography (^32^P). (**b**) As in panel a but in presence of increasing amounts of ADPr. Proteins were incubated for 60 min. (**c**) Substitutions of amino acids in full-length nsP3 shown to affect the catalytic activity of the CHIKV macrodomain were generated, bacterially expressed, purified as His_6_-tagged fusion proteins and subjected to afore MARylated GST-ARTD10cat for the indicated times. Activity was analyzed as described in panel a. (**d**) HA-ARTD1 was expressed in HEK293 cells, immunoprecipitated from cell lysates and automodified by incubation with NAD^+^ in the presence of double stranded oligomers. The PARylated ARTD1 was then incubated with CHIKV-nsP3 for 30 min. PAR chains were evaluated using PAR-specific antibodies on immunoblots. The proteins were stained using Coomassie blue (CB). For control the expression of HA-ARTD1 and α-Tubulin was determined in whole cell lysates (WCL) using immunoblotting. (**e**) Experiments were performed as in panel d and quantified. Mean values ± SD of 3 experiments are shown. (**f**) Quantification of ARTD10 de-MARylation measured at 60 min. Mean values ± SD of 3–5 experiments are shown. CB, Coomassie blue; IP, immunoprecipitation; WCL, whole cell lysate; ^32^P, autoradiogram. ****p < 0.0001; **p < 0.01; ns, not significant when an unpaired *t*-test was applied.

The macrodomain of CHIKV is located at the N-terminus of nsP3, a multi-functional protein that is only partially characterized. To address whether the vMD is also active in full-length nsP3 of CHIKV, His_6_-tagged nsP3 was bacterially expressed and purified and tested for its hydrolase activity (Fig. 7). Comparable to the isolated macrodomain, CHIKV full-length nsP3 was capable of reverting MARylation of GST-ARTD10cat (Fig. 7a). Free ADPr impeded the removal of MARylation in a dose-dependent manner (Fig. 7b), similar to the finding with the isolated vMD (Fig. 3c). Next we determined whether the amino acids, which were identified as important for activity of the isolated vMD (Fig. 5), were also relevant for hydrolase activity of full-length nsP3 (Fig. 7c). Indeed, substitution of V33 with E or Y114 with V resulted in a loss of enzymatic activity. Finally, we tested full-length nsP3 on *in vitro* PARylated ARTD1 and, comparable to the isolated vMD, measured only weak hydrolase activity (Fig. 7d and e). Again, the comparison to the quantified activity of full-length nsP3 on MARylated ARTD10cat revealed a substantial difference to its effect on PARylated ARTD1 (Fig. 7e and f). Together these results demonstrate that the CHIKV macrodomain, either on its own or as part of nsP3, possesses robust hydrolase activity towards several MARylated substrates. In contrast, ARTD1-bound PAR chains are only rather inefficiently hydrolyzed.

### CHIKV macrodomain removes mono-ADP-ribosylation in cells

**Figure 8:**
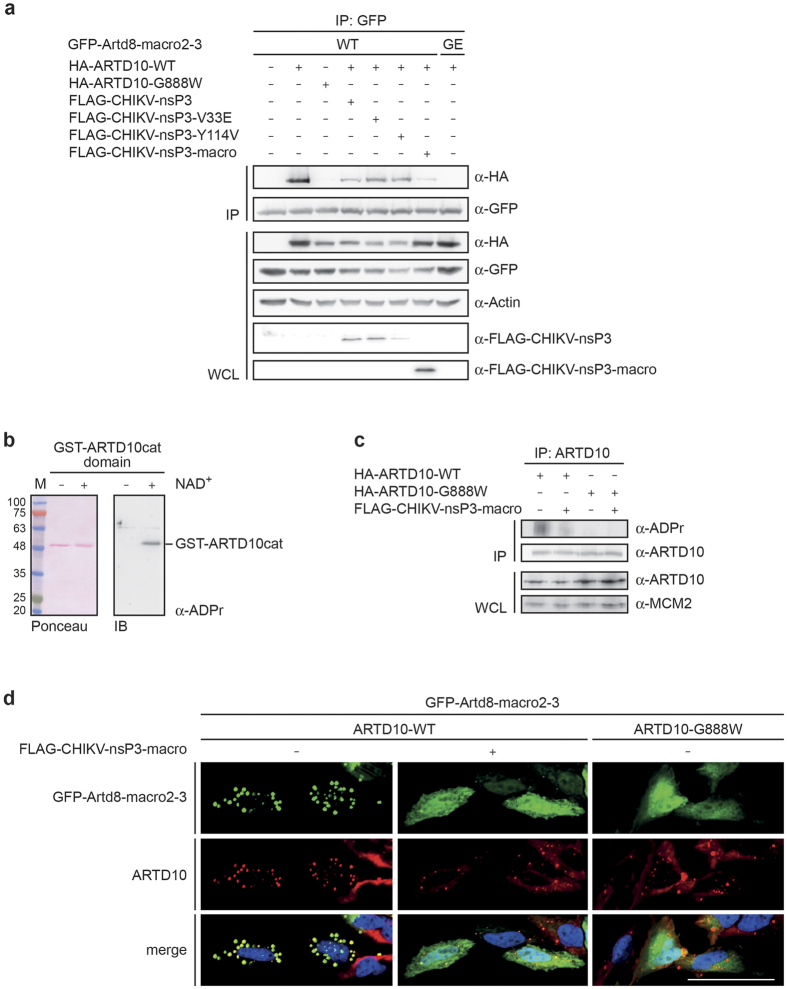
CHIKV macrodomain reverses intracellular MARylation. (**a**) HEK293 cells stably expressing a GFP-Artd8-macro2–3 fusion protein, which reads MARylation, were transiently transfected with plasmids expressing HA-ARTD10-WT or catalytically inactive HA-ARTD10-G888W and the CHIKV macrodomain/nsP3 WT or mutants as indicated. Cells were lysed and GFP-Artd8-macro2–3 immunoprecipitated using anti-GFP antibodies and protein G beads. The amount of co-immunoprecipitated ARTD10 was determined by immunoblotting (mAb 5H11). For expression control the proteins were analyzed in whole cell lysates (WCL) by immunoblotting. (**b**) GST-ARTD10cat was incubated in presence or absence of β-NAD^+^. The proteins were analyzed by immunoblotting using anti-PAR antibodies (Trevigen). For control the blot was stained with Ponceau to visualize GST-ARTD10cat. (**c**) HeLa cells were transiently transfected with expression plasmids for ARTD10-WT, ARTD10-G888W and FLAG-CHIKV-nsP3-macro as indicated. ARTD10 was immunoprecipitated and MARylation determined by immunoblotting using the anti-PAR antibody that is capable of detecting MARylation. For expression control whole cell lysates (WCL) were analyzed. (**d**) HeLa Flp-In T-REx-ARTD10 and ARTD10-G888W cells were transiently transfected with FLAG-CHIKV-nsP3-macro and GFP-Artd8-macro2–3 as indicated and ARTD10 expression was induced. The localization of GFP-Artd8-macro2–3 and ARTD10 was analyzed by confocal microscopy. GFP-Artd8-macro2–3 is shown in green and induced ARTD10 is shown in red. Scale bar: 50 μM. IB, immunoblot; IP, immunoprecipitation; WCL, whole cell lysate.

To address whether the CHIKV macrodomain functions in cells, we co-expressed the vMD or the full-length nsP3 with ARTD10 in cells. As a tool to detect intracellularly MARylated proteins we used GFP-fusion proteins of macrodomains 2 and 3 of Artd8 (GFP-Artd8-macro2–3), previously identified to function as binding modules specific for MARylated proteins^10^, that were stably expressed in HEK293 cells. In co-immunoprecipitation (co-IP) experiments using GFP-specific antibodies, we measured bound ARTD10-WT and ARTD10-G888W, a catalytically inactive mutant, as a means to assess MARylation (Fig. 8a). As expected, GFP-Artd8-macro2–3 interacted with MARylated ARTD10-WT but poorly with ARTD10-G888W. Co-expression of the CHIKV macrodomain or nsP3 hampered binding of ARTD10 to GFP-Artd8-macro2–3, whereas co-expression of mutants with reduced hydrolase activity (nsP3-V33E, nsP3-Y114V) showed little effect. Because binding between GFP-Artd8-macro2–3 and ARTD10 is dependent on MARylation of ARTD10, these experiments strongly indicated that the CHIKV macrodomain was capable of reverting MARylation of ARTD10 in cells. Nonetheless, the expression of the vMD might have unanticipated consequences. Therefore, we wanted to apply an additional experimental set-up. For this we made use of an antibody initially made against PAR, which we found to also interact with the automodified, MARylated catalytic domain of ARTD10 (Fig. 8b). Thus in addition to the co-IP experiments with GFP-Artd8-macro2–3, we analyzed MARylation of immunoprecipitated ARTD10 using the α-ADPr antibody. We observed a signal of overexpressed and immunoprecipitated ARTD10-WT but not of ARTD10-G888W from HeLa cell lysates (Fig. 8c). When the CHIKV macrodomain was co-expressed, MARylation of ARTD10-WT was reduced (Fig. 8c). In line with these experiments co-localization of GFP-Artd8-macro2–3 with ARTD10-WT in cells was reduced upon co-expression of the CHIKV macrodomain (Fig. 8d). While GFP-Artd8-macro2–3 was almost exclusively associated with ARTD10-WT dots^10^,^44^, it relocated throughout the cell when the vMD was co-expressed. This was comparable to the distribution of GFP-Artd8-macro2–3 in the presence of the catalytically inactive ARTD10-G888W. Of note, the ARTD10-WT dots showed some co-localization with GFP-Artd8-macro2–3 in the presence of the CHIKV macrodomain, probably because the vMD was unable to remove all MARylation. Together these findings provide strong support for intracellular hydrolase activity of the CHIKV macrodomain towards MARylated ARTD10.

## Discussion

Stimulation of cells by IFNα defined *ARTD10* and *ARTD8* as target genes of this cytokine, as documented by both elevated mRNA and protein expression (Fig. 1). IFNα represents the first line response against invading viruses and triggers a robust and complex antiviral cellular program by inducing more than 300 IFN-stimulated genes (ISGs)^45^,^46^. Thus these two mono-ARTDs are ISGs, consistent with previous studies demonstrating that the expression of these genes is induced in response to pathogens^3^,^17^. The induction of *ARTD8* and *ARTD10* as measured in time-course experiments occurred with some delay suggesting that they are not direct IFNα/ISGF3 (STAT1-STAT2-IRF9 heterotrimeric complex) target genes or that the cooperativity with additional signals is relevant for induced expression.

The delayed expression indicates a role of ARTD8 and ARTD10 in balancing the immune response as part of a feedback loop. Both mono-ARTDs show N-terminally localized putative RNA recognition motifs that might be relevant to recognize foreign RNA of invading pathogens, which could function as a signal to further develop an appropriate immune response^1^. In that respect it is worth noting that ARTD10 interferes with NF-κB signaling, a pathway that is critical for shaping immune cell functions^40^. Additional findings support a role of ARTDs in antiviral defense. These include the antiviral role of ARTD13 and the proposed evolution of several ARTDs in response to pathogens^17^,^23^. Moreover, several mono-ARTDs are associated with stress granules, structures that have been implicated in the control of replication of some viruses^47^. Furthermore, and in more general terms, PTMs, including phosphorylation and ubiquitinylation, which fulfill essential roles in controlling protein activities in cells, are manipulated by pathogens to promote infection and propagation^48^. Thus the information we have so far about mono-ARTDs suggests that MARylation is a likely target of viruses. This is now supported by our findings that vMDs function as mono-ADP-ribosylhydrolases.

The vMDs of CHIKV, ONNV, SINV, VEEV, HEV, and FIPV all possess de-MARylating activity, while they are not efficient in hydrolyzing PAR chains (Figs 2, 3, 4, 7 and 8, Supplementary Figs S2, S4 and S5). *In silico* and mutational analysis as well as subsequent biochemical experiments with macrodomain mutants and the CHIKV-nsP3 full-length support this novel enzymatic activity for vMDs (Figs 5, 6 and 7). All tested vMDs appear to elicit broad catalytic activity as they de-MARylate automodified ARTD7, ARTD8, ARTD10 and MARylated NEMO, an ARTD10 substrate^40^. Moreover, ARTD10, and possibly the other substrates as well, is modified at multiple sites^2^. This enhances substrate complexity supporting our interpretation of broad catalytic activity. This is also consistent with the findings discussed above that multiple mono-ARTDs are potentially associated with antiviral activity and thus vMDs are expected to be capable of de-MARylating multiple substrates. We demonstrated that ARTD10 is also de-MARylated in cells suggesting that vMDs can function intracellularly (Fig. 8). What the relevant substrates are that need to be de-MARylated in virally infected cells remains to be determined. Our understanding about MARylated substrates is very limited at present and only few proteins have been demonstrated to be modified in cells^10^,^11^,^40^. Thus it will be interesting to define relevant targets of the catalytic activities of vMDs and to understand the biological consequences of MARylation and de-MARylation and their potential importance for virus propagation.

Some vMDs have been described to interact with PAR chains^25^,^49^,^50^. Taking the overall highly positive electrostatic surface potential of vMDs into account^25^, binding to negatively charged molecules like PAR is not unexpected and specificity is a concern. Despite this interaction, we could not detect substantial hydrolase activity of the vMDs towards PAR chains synthesized on ARTD1 (Figs 4 and 7, Supplementary Figs S4 and S5). Of note is that the linkage between the protein and its proximal ADPr, an ester bond with an acidic amino acid, is different from the ribose-ribosyl *O*-glycosidic bond found within PAR chains. Thus it is likely that different catalytic mechanisms are needed to either trim PAR chains or to cleave the bond between the amino acid side chain and ADPr, being it a PAR chain or a single ADPr unit. Distinguishing between the latter will depend on whether the vMDs are able to accommodate the amino acid – ribose ester bond in the active center despite one or more additional ADPr units bound to the amino acid linked ADPr, as in PAR chains. Looking at the crystal structure of the CHIKV macrodomain in complex with ADPr it might be possible that this vMD can interact with a PAR chain^25^. However, our findings suggest that none of the tested vMDs, including the one of CHIKV, has strong activity against PARylated ARTD1. This argues that the amino acid – ribose ester bond of PAR chains is less well accessible and/or positioned in the active center compared to the respective bond in a MARylated substrate. As a consequence, complete PAR chains are less efficiently released than mono-ADPr from substrates.

While our work was under consideration, a report was published suggesting that vMDs hydrolyze both MARylation and PARylation^35^. We note that the results suggesting activity against PAR chains are not easy to interpret due to significant background in some experiments^35^. Moreover, ARTD1 was ^32^P-ADP-ribosylated under low NAD^+^ conditions, which have been shown by others to also result in substantial MARylation in addition to PARylation^12^. Thus, the release of radioactivity from labeled ARTD1 might be at least in part due to the hydrolysis of MARylation. This is consistent with our findings that all vMDs tested are significantly more active towards MARylation compared to PARylation (Figs 4 and 7, Supplementary Figs S4 and S5). An additional aspect that is worth considering relates to the findings that ARTD1 has been reported to not only modify acidic amino acids but also lysines^51^,^52^. Thus it might be that the vMDs remove PAR chains from glutamate or aspartate but not from lysine, as the biochemical reaction would be expected to be rather distinct. Together, the findings strongly suggest that these vMDs have evolved specifically as de-MARylating enzymes.

Based on *in silico* analysis of the CHIKV macrodomain, amino acids N24, V33 and Y114 were suggested to be important for ligand binding and thus most likely for catalytic activity (Figs 5 and 6). Up to date a precise and validated catalytic mechanism that explains the removal of the protein-proximal ADPr is lacking^11^,^12^,^14^. Our experimental results obtained with CHIKV macrodomain mutants, which were based on *in silico* alanine scanning and calculating Amber scores, are also in agreement with molecular dynamic simulations proposing N24, V33 and Y114 of the CHIKV macrodomain to be important for enzyme-substrate interaction and N24 and Y114 for catalysis^39^. Having shown that the inactivating mutations, including V33E and Y114V, do not affect the overall structure of the CHIKV macrodomain (Supplementary Fig. S6b), it will now be important to address their biological relevance for viral replication. In that respect the reported findings on HEV macrodomain mutations are worth discussing^35^. A strong effect on replication is observed with a triple mutation (G48S/G49S/G50A), which shows reduced binding to PAR chains. It will be interesting to determine whether this is due to reduced catalytic activity or altered structure of the macrodomain. The latter could have effects on the overall function of ORF1 or nsP3. Indeed, the macrodomain of HEV interacts with other non-structural proteins, which has been suggested to be of functional relevance for viral replication^53^, that might be abolished by structural changes. Of note is also that the G48/G49/G50 motif in HEV is conserved in other vMDs (e.g. SINV (GEG), VEEV (GGG), CHIKV (GDG), ONNV (GDG), and FIPV (VGG)). Within this motif the respective Aspartate 31 of the CHIKV macrodomain was considered in our *in silico* analysis but substitution by alanine resulted in a highly negative ∆∆G value, suggesting that this change would rather stabilize the interaction with ADP-ribose. Therefore, we did not further evaluate D31 (Fig. 5c).

In conclusion, our findings demonstrate that several macrodomains of different genera of (+)ssRNA viruses possess mono-ADP-ribosylhydrolase activity against diverse substrates. This suggests, together with the IFNα responsiveness of several mono-ARTDs as well as published findings, that MARylation is a PTM that is relevant for the innate immune response. Our previous studies on ARTD10 and ARTD8 substrates suggest that multiple signaling pathways are regulated by MARylation^11^,^40^,^54^. However, for most of the potential substrates the functional consequences of MARylation are not known and more strinkingly most of the substrates of MARylation are probably not even identified until now. Also, how mono-ARTDs are controlled, beyond effects on gene transcription, is poorly understood. Taken together, this highlights MARylation as a PTM of emerging relevance with the potential for an important role in viral defense or more generally in host-pathogen conflicts.

## Methods

### Cell lines and cell culture

HeLa, U2OS, THP-1, HEK293, HEK293 Flp-In T-REx-GFP-Artd8-macro2–3 and HeLa Flp-In T-REx-ARTD10 and -ARTD10-G888W cells^55^ were cultivated in DMEM supplemented with 10% heat-inactivated fetal calf serum (FCS) or in RPMI with 10% FCS (THP-1) at 37 °C in 5% CO_2_. Transfections were performed using the calcium phosphate precipitation technique. HEK293 Flp-In T-REx cells were transfected with pcDNA5/FRT/TO-Artd8-macro2–3 or a plasmid containing the respective binding dead mutant (Artd8-macro2-G1055E-macro3-G1268E) and pOG44 (Invitrogen) and selected using 5 μg/mL Blasticidin S (Invivogen) and 200 μg/mL Hygromycin B (Invivogen).

### Reagents and antibodies

The following reagents were used: β-NAD^+^ (Sigma), ^32^P-NAD^+^ (Perkin Elmer), interferon α (Peprotech), lipopolysaccharid (LPS) of *E. coli* (Sigma), 12-O-Tetradecanoylphorbol-13-acetate (PMA) (Sigma), protease inhibitor cocktail (PIC) (Sigma), H_2_O_2_ (Merck KGaA), Glutathione-sepharose (Sigma), TALON metal affinity resin (BD Bioscience), ADPr (Adenosine 5′ diphosphoribose sodium-salt, Sigma), anti-HA (3F10, Roche), anti-HA (Covance), anti-PAR (Trevigen), anti-α-Tubulin (Sigma), anti-ARTD10 (5H11)^2^, anti-ARTD10 purified rabbit antibodies^10^, anti-Actin (C4, MP Biomedicals), anti-FLAG (Sigma), anti-GFP (Rockland), anti-MCM2 (N-19, Santa Cruz), goat anti-rat IgG (H + L) secondary antibody Alexa Fluor 555 conjugate (Invitrogen), anti-rabbit-HRP (Jackson Immunoresearch), anti-mouse-HRP (Jackson Immunoresearch), anti-rat-HRP (Jackson Immunoresearch). Rabbit polyclonal, purified ARTD8-specific antibodies were generated against the peptide NLVSDKIPKAKDTQG (aa 1193–1207).

### Reporter gene assay

To measure *ARTD10* promoter activity U2OS cells were seeded in 12-well plates and transiently transfected with the pGL3basic-ARTD10 promoter reporter gene construct. To normalize transfection efficiency a β-galactosidase expressing plasmid was co-transfected. 24 h after transfection cells were stimulated with IFNα for 16 h and subsequently harvested using extraction buffer (5 mM Tris, pH 7.8; 0.4 mM EDTA; 2% glycerol; 0.2% Triton X-100; 10 mM DTT). Luciferase activities were normalized to β-galactosidase activities.

### Quantitative real-time PCR and Immunoblot analysis

HeLa or U2OS cells were stimulated with IFNα (180 U/mL). THP-1 cells were differentiated with PMA (200 ng/mL) for 24 h before stimulation with LPS (100 ng/ml). Total RNA was isolated using the High Pure RNA Isolation Kit (Roche) according to the manufacturer’s protocol. Reverse transcription was performed with 1 μg of RNA using the QuantiTect Reverse Transcription Kit (QIAGEN). *ARTD8* and *ARTD10* expression was analyzed by quantitative real-time PCR (qRT-PCR) using QuantiTect Primer Assays (QIAGEN). In each experiment, mRNA expression of the gene of interest was normalized to *GUS* (QuantiTect Primer Assays, QIAGEN).

For protein analysis cells were treated as above and harvested in RIPA buffer (10 mM Tris, pH 7.4; 150 mM NaCl; 1% NP-40; 1% DOC; 0.1% SDS; PIC). Cellular debris was removed by centrifugation for 25 min at 4 °C. Lysates were fractionated using SDS-PAGE and analyzed by immunoblotting.

### Cloning and mutagenesis

The cDNA encoding a 1048 bp long fragment of the human *ARTD10* promoter (−532 to +491, relative to the start of transcription) was generated from HeLa DNA using sequence specific primers harboring restriction sites for *HindIII* and *NcoI*. The fragment was cloned in front of a luciferase reporter gene into the pGL3basic reporter gene construct.

GST-fusion proteins of ARTD10cat have been described previously^2^. The cDNAs encoding for the catalytic domains of ARTD7 (N459 - A656) and ARTD8 (K1600–K1800) as well as ARTD8-macro1–3 were generated from plasmids obtained from H. Schüler (Stockholm) and cloned into pGEX-4T1 or pEGFP-C1 using the gateway cloning strategy. Bacterial expression constructs for His_6_-tagged fusion proteins of the viral macrodomains of CHIKV, FIPV, VEEV, HEV-XDA4 and HEV-XDD12 were obtained from B. Coutard (Marseille). Plasmids encoding the viral macrodomains of SINV and ONNV were generated by LIC and cloned into pNIC-Bsa4. Clones expressing viral macrodomains, CHIKV-nsP3 and mutants thereof were generated by gateway cloning. Mutants were generated using standard mutagenesis procedures and confirmed by sequencing. pcDNA3-HA-ARTD1 was a kind gift from M. Hottiger (Zürich).

### Purification of His- and GST-tagged fusion proteins

His_6_- and GST-tagged fusion proteins were expressed in *E. coli* BL-21 except for HEV-XDA4 and HEV-XDD12 macrodomains, which were expressed in *E. coli* XL-10. Purification of the recombinant proteins was achieved using affinity chromatography on either glutathione-sepharose for GST-fusion proteins or TALON metal affinity resin for His_6_-fusion proteins according to standard protocols.

### Hydrolase assay with immunoprecipitated ARTD1

HEK293 cells were transiently transfected with a plasmid encoding HA-ARTD1. The cells were harvested in TAP lysis buffer (50 mM Tris, pH 7.5; 150 mM NaCl; 1 mM EDTA; 10% glycerol; 1% NP-40; 1 mM DTT; PIC) and the lysates were centrifuged at 4 °C for 25 min. HA-ARTD1 was immunoprecipitated with 10 μl of anti-HA (3F10 or Covance) antibodies and protein G beads at 4 °C for 1 h. Thereafter the beads were washed in TAP lysis buffer and in reaction buffer (50 mM Tris pH 8.0; 0.2 mM TCEP; 4 mM MgCl_2_; 0.2% NP-40). ADP-ribosylation was carried out in 30 μl reaction buffer supplemented with 50–500 μM β-NAD^+^, depending on the age of the ^32^P-NAD^+^, and 5 pmol annealed double stranded oligomers (5′-CACCGTGTCAGGACCACTAGCCTCT-3′) at 30 °C for 30 min. Reactions were stopped by washing in reaction buffer. Then 1 μg of the respective purified His_6_-tagged vMD was added and incubated at 30 °C for 60 min. Reactions were analyzed by SDS-PAGE and immunoblotting. For endogenous ARTD1 PARylation HeLa cells were transiently transfected with a plasmid expressing HA-ARTD1. Prior to harvesting cells were treated with 1 mM H_2_O_2_ for 7 min to promote PARylation. For control cells were incubated with 10 μM Olaparib for 2 h. ARTD1 was immunoprecipitated, treated with vMDs, and analyzed as described above. The PAR signals were quantified using the “Image J” software (NIH, Bethesda, USA) and the significance analyzed using an unpaired *t*-test.

### Hydrolase assay with purified recombinant proteins

1 μg of GST-fusion proteins with the catalytic domains of ARTD7, ARTD8, and ARTD10 and with NEMO were bound to glutathione-sepharose beads in pulldown buffer (100 mM Tris, pH 7.6, 250 mM NaCl, 50 mM KCl, 5 mM MgCl_2_, 0.5% NP-40, 0.1% Triton X-100) at 4 °C for 60 min. The beads were washed and equilibrated in reaction buffer. ADP-ribosylation was carried out in 30 μl reaction buffer with 50 μM β-NAD^+^ and 1 μCi ^32^P-NAD^+^ at 30 °C for 30 min. Reactions were stopped by washing with reaction buffer. For hydrolase assays 1 μg of purified His_6_-tagged vMD was added and incubated at 30 °C for the times indicated. Reactions were fractionated by SDS-PAGE and the gels were stained with Coomassie blue. The incorporated ^32^P-label was detected on X-ray films.

### Analysis of released products from hydrolase assays by thin layer chromatography

ADP-ribosylation reactions were carried out in presence of ^32^P-NAD^+^. After the subsequent hydrolase assays, the supernatants were removed from the beads and subjected to thin layer chromatography (TLC) to analyze the released products. The samples were spotted onto PEI-F cellulose plates (20 cm × 20 cm, Merck) and developed in 0.3 M LiCl and 0.9 M acetic acid and evaluated by exposure to X-ray films.

### Immunoprecipitations and immunofluorescence microscopy

Stable HEK293 Flp-In T-REx-GFP-Artd8-macro2–3 cells were cotransfected with constructs expressing HA-ARTD10-WT or HA-ARTD10-G888W and CHIKV-nsP3-macro or CHIKV-nsP3 and mutants as indicated. After 24 h expression of GFP-Artd8-macro2–3 was induced by the addition of doxycycline (500 ng/mL) over night. Thereafter cells were harvested and lysed in TAP lysis buffer and the lysates cleared. GFP-Artd8-macro2–3 was immunoprecipitated using GFP-specific antibodies and protein G beads at 4 °C for 1 h. Co-immunoprecipitated ARTD10 was detected by SDS-PAGE and immunoblotting.

HEK293 cells were transiently transfected with plasmids encoding ARTD10-WT or ARTD10-G888W and CHIKV-nsP3-macro. Cell lysates were generated in RIPA buffer and ARTD10 immunoprecipitated using specific antibodies. Bound proteins and their MARylation were analyzed by immunoblotting.

Stable HeLa Flp-In T-REx-ARTD10 and -ARTD10-G888W were transiently transfected with plasmids encoding FLAG-CHIKV-nsP3-macro and GFP-Artd8-macro2–3. ARTD10 expression was induced by doxycycline (500 ng/mL) over night. Cells were fixed with 3.7% paraformaldehyde, permeabilized with 40 μg/mL Digitonin in PBS and blocked by incubation with PBS containing 3% BSA at room temperature for 30 min. Fixed cells were stained for ARTD10 with mAb 5H11, washed and incubated with anti-rat-Alexa Fluor 555 secondary antibodies in PBS containing 1% BSA. Nuclei were stained with 1 μg/mL Hoechst 33258 (SigmaAldrich) diluted in ddH_2_O. Samples were mounted with Mowiol 4–88 (SigmaAldrich).

Images were captured using a Zeiss LSM 710 Confocal Laser Scanning Microscope equipped with an AxioCam (Zeiss) and a C-Apochromat 40x water immersion objective. For all pictures the pinhole was set to 1 airy unit and a resolution of 1024 × 1024 pixels was chosen. The EGFP fluorochrome was excited at a wavelength of 488 nm (emission maximum: 509 nm) by an argon laser with 30 mW output and detected using a 488 nm single channel PTM and a 505–539 nm bandpass filter. The fluorochrome Alexa Fluor 555 was excited by a helium-neon-laser with 1 mW output at a wavelength of 543 nm (emission maximum: 565 nm) and detected using a 543 main beam splitter and a spectral META-detector of 552–627 nm. The nuclei stained by Hoechst were excited by a UV-laser at a wavelength of 352 nm (emission maximum: 455 nm) and detected using a main beam splitter and a bandpass filter of 454–553 nm.

### Poly-acrylamide gel electrophoresis (PAGE), Computational and circular dichroism analysis

See Supplementary Information.

## Additional Information

**How to cite this article:** Eckei, L. *et al*. The conserved macrodomains of the non-structural proteins of Chikungunya virus and other pathogenic positive strand RNA viruses function as mono-ADP-ribosylhydrolases. *Sci. Rep.* **7**, 41746; doi: 10.1038/srep41746 (2017).

**Publisher's note:** Springer Nature remains neutral with regard to jurisdictional claims in published maps and institutional affiliations.

## Supplementary Material

Supplementary Information
