# Supplementary material for: The conserved macrodomains of the non-structural proteins of Chikungunya virus and other pathogenic positive strand RNA viruses function as mono-ADP-ribosylhydrolases
Source: Sci Rep. 2017 Feb 2;7:41746. doi: 10.1038/srep41746 (PMC5288732; doi:10.1038/srep41746)
Supplement: Supplementary Information [file srep41746-s1.pdf]

Supplementary information Eckei et al.

The conserved macrodomains of the non-structural proteins of Chikungunya virus and other pathogenic positive strand RNA viruses function as mono-ADP-ribosylhydrolases

Laura Eckei[1,7], Sarah Krieg[1,7], Mareike Bütepage[1], Anne Lehmann[1], Annika Gross[1,8], Barbara Lippok[1], Alexander Grimm[2], Beate Kümmerer[3], Giulia Rossetti[4,5,6], Bernhard Lüscher[1,9,10], Patricia Verheugd[1,9,10]

[1]Institute of Biochemistry and Molecular Biology, Medical School, RWTH Aachen University, 52057 Aachen, Germany

[2]Institute of Biotechnology, RWTH Aachen University, 52074 Aachen, Germany

[3]Institute of Virology, University of Bonn Medical Centre, 53127 Bonn, Germany

[4]Computational Biomedicine, Institute for Advanced Simulation IAS-5 and Institute of Neuroscience and Medicine INM-9, Forschungszentrum Jülich, 52425, Jülich, Germany

[5]Jülich Supercomputing Centre, Forschungszentrum Jülich, 52425, Jülich, Germany

[6]Department of Oncology, Hematology and Stem Cell Transplantation, Medical School, RWTH Aachen University, Aachen, Germany

[7]These authors contributed equally to this work.

[8]Present address: Department of Internal Medicine III, Medical School, 52057 Aachen, Germany

[9]Equally contributing senior authors

[10]Correspondence should be addressed to B.L. (luescher@rwth-aachen.de) and P.V. (pverheugd@ukaachen.de).

Supplementary methods

Analysis of released products from hydrolase assays by poly-acrylamide gel electrophoresis (PAGE)

Hydrolase assays were carried out with immunoprecipitated HA-ARTD1 that was labeled in the presence of 100 µM β-NAD^+, 5 pmol annealed double stranded oligomers and 1 µCi ^{32}P-NAD^+ at 30°C for 30 min. Following the incubation step the supernatants were removed from the beads. The material bound to the beads and a fraction of the supernatant were used for SDS-PAGE analysis and Coomassie blue (CB) staining to visualize the amount of immunoprecipitated HA-ARTD1 and the vMDs that were included in the reactions. Furthermore the dried gels were subjected to autoradiography (^{32}P) to depict the remaining automodification of HA-ARTD1. The supernatant and thus the released products of the hydrolase assays were further subjected to PAGE to determine polymer length. The PAGE was carried out as previously described [1]. Chemical detachment and precipitation of the modifications were not necessary and therefore the reaction's supernatants were simply dried in a Speed Vac and resuspended in 5 µl of loading buffer (50% urea w/v, 25 mM NaCl, 4 mM EDTA, 0.02% xylene cyanol, 0.02% bromophenol blue).

In silico alanine scanning

The ABS-Scan web-server [2] systematically evaluates amino acids for their importance in protein-ligand interactions by *in silico* alanine scanning. The crystal structure of the Chikungunya macrodomain in complex with ADP-ribose (PDBID 3GPO) was used as starting structure [3]. A distance cut-off of 5 Å was chosen to define the binding site around the ADP-ribose. For each residue within the cut-off, all side chain atoms beyond $C_β$ were removed and the missing hydrogen was added, obtaining an alanine side chain. Modeler library was used on all selected residues, coupled with steps of energy minimization to ensure that no steric clashes occur between protein and ligand atoms [4]. The analysis and results derived from alanine scanning mutagenesis relies on two assumptions: (i) The introduced point mutation does not drastically change the structure of the protein and (ii) the mode of ligand interaction is unchanged [2]. The structural quality of the generated protein structures was estimated through Discrete Optimized Protein Energy (DOPE) score [5], while the

energetics of a protein-ligand complex was scored by using Autodock 4.1 forcefield [6]. The contribution of a specific amino acid is determined by the difference in interaction score of mutant and wild-type protein (ΔΔG value). From this procedure three relevant residues for ADP-ribose binding were identified, namely N24, V33 and Y114 (hot-spot residues, hereafter).

Computational mutagenesis

The three residues identified in the *in silico* alanine scanning were systematically exchanged against all other 19 amino acids by using the Swiss-PdbViewer package [7]. The energetics of the protein-ligand complex of all protein variants of the three hot-spot residues (N24, V33 and Y114) were evaluated by the Amber score. Amber score implements molecular mechanics Generalized Born/surface area simulations with traditional general Amber force field for ligand molecules [8]. The interaction between the ligand and the protein is represented by electrostatic and van der Waals energy terms, and the solvation energy is calculated using Generalized Born solvation model. In this protocol it is implicitly assumed that point mutations in the protein do not significantly affect the conformation of the mutated protein. During Amber score calculation, the input coordinates of the different amino acids for each hot-spot residue are minimized using the conjugate gradient method to remove poor contacts. This is followed by molecular dynamics simulation (Langevin dynamics at constant temperature), and a short minimization to obtain the final energy of the system. The Amber score is calculated as E(Complex) - [E(Protein) + E(Ligand)]. The entropic contribution is supposed to be constant in the mutated and wild type structure considering their similarity and was therefore not calculated, as discussed previously [9]. From these procedure two mutants for each hot-spot residue were identified to strongly destabilize the protein-ligand complex without introducing dramatic structural changes to the protein. These are N24Y, N24R, V33E, V33F, Y114V, and Y114W.

Circular dichroism analysis

The buffer of the bacterially expressed and purified His_6-tagged fusion proteins of the CHIKV-nsP3 macrodomain WT and mutants was exchanged for CD buffer (10 mM potassium phosphate pH 7.5, 100 mM $(NH4)_2SO_4$, 10% glycerol) using 10 kDa MWCO centrifugal filter devices (Amicon Ultra-0.5, Merck Millipore, Billerica, MA,

USA) by centrifugation (14,000 g, 4°C, 15 min). Afterwards protein concentrations were determined using the "Bio-Rad DC™ Proteinassay Kits" (Bio-Rad) and adjusted to 0.5 mg/mL with CD buffer. Subsequently the protein purity was assessed by SDS-PAGE and the gels were stained with Coomassie blue (CB).

The sample volume in each circular dichroism (CD) measurement was 140 µL. Each sample was transferred onto a Hellma® SUPRASIL cuvette (Hellma GmbH & Co. KG) with a pathlength of 0.5 mm. The sample analysis was carried out in a triplicate scan from 195 to 240 nm at room temperature with the Olis SDM 17 CD (Olis). CD buffer measurements provided the baseline and the scans were averaged. The CD spectra were smoothed employing the Savitzky-Golay filter (Olis Global Works software package) with a filter size of 11 [10].

1 Panzeter, P. L. & Althaus, F. R. High resolution size analysis of ADP-ribose polymers using modified DNA sequencing gels. *Nucleic Acids Res* **18**, 2194 (1990).

2 Anand, P., Nagarajan, D., Mukherjee, S. & Chandra, N. ABS-Scan: In silico alanine scanning mutagenesis for binding site residues in protein-ligand complex. *F1000Res*, doi:papers2://publication/uuid/90925C21-45FE-4D12-AD5F-248716BA8AB2 (2013).

3 Malet, H. *et al.* The Crystal Structures of Chikungunya and Venezuelan Equine Encephalitis Virus nsP3 Macro Domains Define a Conserved Adenosine Binding Pocket. *Journal of Virology* **83**, 6534-6545, doi:10.1128/jvi.00189-09 (2009).

4 Sali, A. & Blundell, T. L. Comparative protein modelling by satisfaction of spatial restraints. *J. Mol. Biol.* **234**, 779-815, doi:papers2://publication/doi/10.1006/jmbi.1993.1626 (1993).

5 Shen, M.-y. & Sali, A. Statistical potential for assessment and prediction of protein structures. *Protein Science* **15**, 2507-2524, doi:papers2://publication/doi/10.1110/ps.062416606 (2006).

6 Huey, R., Morris, G. M., Olson, A. J. & Goodsell, D. S. A semiempirical free energy force field with charge-based desolvation. *J. Comput. Chem.* **28**, 1145-1152, doi:papers2://publication/doi/10.1002/jcc.20634 (2007).

7 Schwede, T., Kopp, J., Guex, N. & Peitsch, M. C. SWISS-MODEL: An automated protein homology-modeling server. *Nucleic Acids Research* **31**, 3381-3385, doi:papers2://publication/uuid/B4DC05DC-0F49-4AF0-A1C4-3C831B5A5D6E (2003).

8 Wang, J., Wolf, R. M., Caldwell, J. W., Kollman, P. A. & Case, D. A. Development and testing of a general amber force field. *J. Comput. Chem.* **25**, 1157-1174, doi:papers2://publication/doi/10.1002/jcc.20035 (2004).

9 Massova, I. & Kollman, P. A. Computational Alanine Scanning To Probe Protein−Protein Interactions: A Novel Approach To Evaluate Binding Free

Energies. *J. Am. Chem. Soc.* **121**, 8133-8143, doi:papers2://publication/doi/10.1021/ja990935j (1999).
10 Gorry, P. A. General least-squares smoothing and differentiation by the convolution (Savitzky-Golay) method. *Analytical chemistry* **62**, 570-573 (1990).

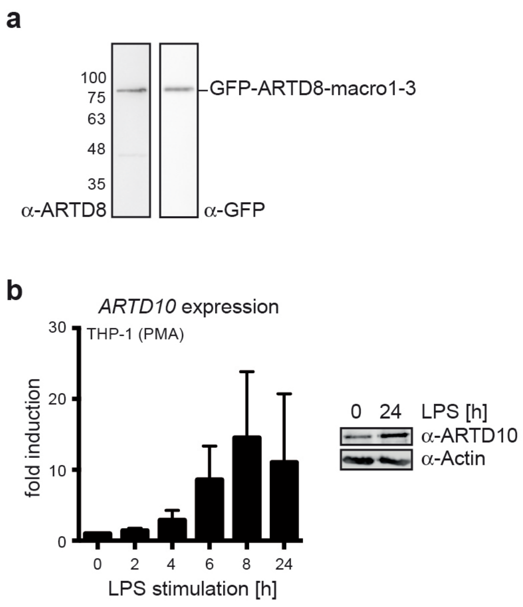

Supplementary Figure S1

(**a**) HeLa cells were transiently transfected with an expression plasmid for GFP-ARTD8-macro1-3. Cells were lysed and lysates separated by SDS-PAGE and immunoblotted for testing the ARTD8 antibody. The antibody, generated against a peptide derived from the sequence located between macrodomain 2 and 3, was able to detect GFP-ARTD8-macro1-3. As control the fusion protein was also detected by a GFP antibody.

(**b**) PMA differentiated THP-1 cells were stimulated with LPS for the indicated times and the mRNA expression of *ARTD10* was determined using RT-qPCR (mean values ± SD of three experiments) and ARTD10 protein was evaluated by immunoblotting using mAb 5H11.

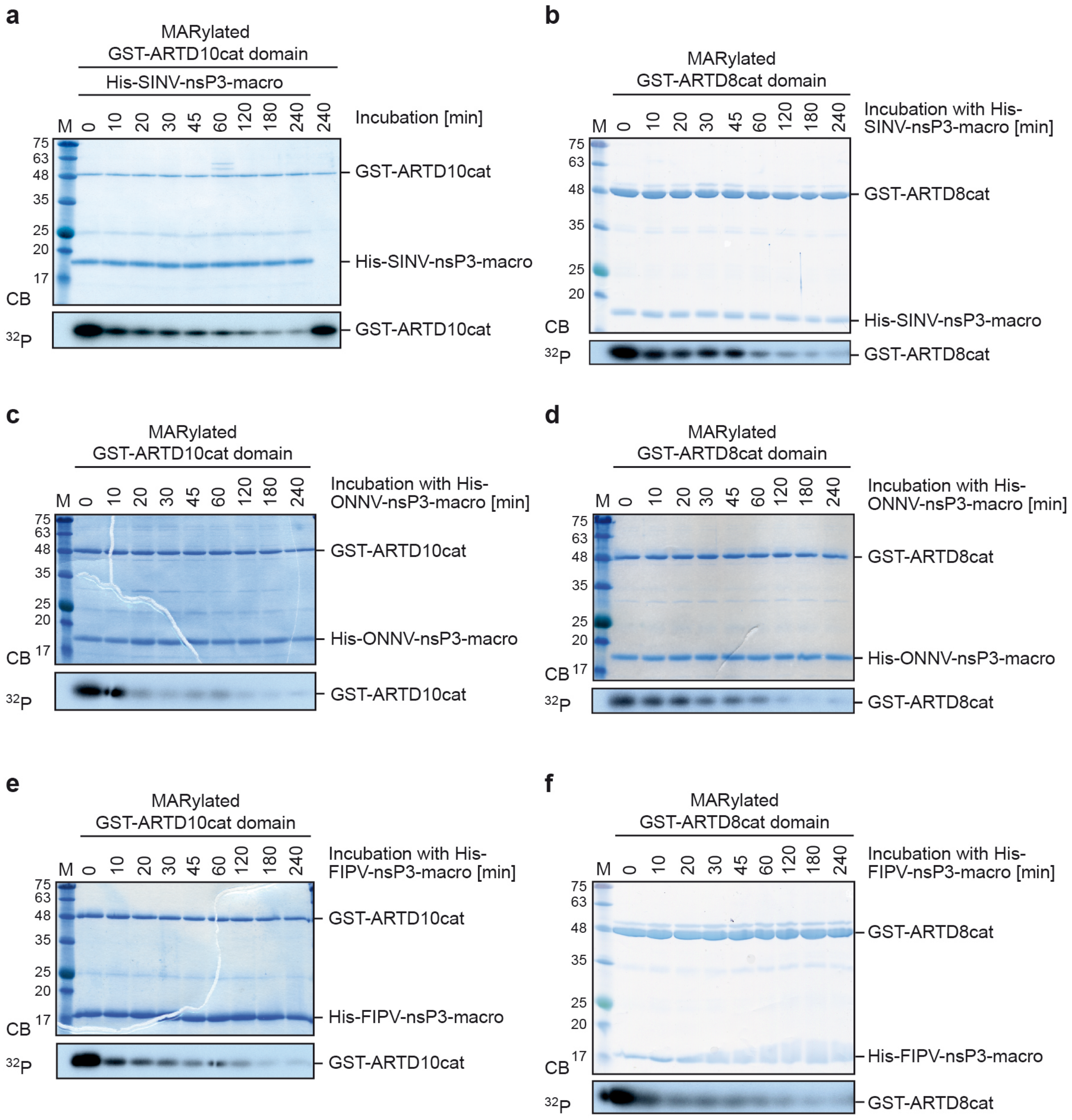

Supplementary Figure S2

(**a**) The GST-ARTD10cat domain was automodified in the presence of ^{32}P-NAD^{+}. The proteins were then incubated with SINV-nsP3 macrodomain for the indicated times. The proteins were stained using Coomassie blue (CB) and the radioactivity associated with the different substrates was assessed by autoradiography (^{32}P).

(**b**) As in panel a but with the automodified catalytic domain of ARTD8 as substrate.

(**c**) As in panel a but with the ONNV-nsP3 macrodomain.

(**d**) As in panel c but with the automodified catalytic domain of ARTD8 as substrate.

(**e**) As in panel a but with the FIPV-nsP3 macrodomain.

(**f**) As in panel e but with the automodified catalytic domain of ARTD8 as substrate.

CB, Coomassie blue; ^{32}P, autoradiogram.

a

	TARG1	PARG	HEV-ORF1-macro	FIPV-nsP3-macro	MHV-nsP3-macro	ARTD8-macro2	ARTD8-macro3	SINV-nsP3-macro	VEEV-nsP3-macro	CHIKV-nsP3-macro	ONNV-nsP3-macro	macroH2A1.1	AF1521	ARTD8-macro1	MacroD1	MacroD2
TARG1		13.04	13.68	13.74	13.74	15.86	17.04	13.18	16.28	13.95	13.18	14.86	15.44	15.89	11.27	11.72
PARG	13.04		13.77	10.07	12.16	11.80	14.29	11.84	14.47	15.79	18.42	15.27	16.09	10.00	13.40	16.54
HEV-ORF1-macro	13.68	13.77		19.84	20.00	19.71	14.84	32.48	29.91	29.06	29.06	17.38	15.83	20.42	21.19	16.49
FIPV-nsP3-macro	13.74	10.07	19.84		31.71	16.77	17.48	24.82	25.55	21.90	23.36	15.09	23.75	27.04	24.84	27.10
MHV-nsP3-macro	13.74	12.16	20.00	31.71		16.77	18.44	23.53	25.74	23.53	22.79	15.92	24.05	22.78	21.57	26.62
ARTD8-macro2	15.86	11.80	19.71	16.77	16.77		22.56	16.99	13.73	16.99	16.99	22.22	17.82	24.72	19.21	20.34
ARTD8-macro3	17.04	14.29	14.84	17.48	18.44	22.56		14.39	19.42	17.99	18.71	20.24	27.33	23.64	23.90	25.16
SINV-nsP3-macro	13.18	11.84	32.48	24.82	23.53	16.99	14.39		53.12	56.88	55.62	17.11	21.71	19.74	30.14	33.56
VEEV-nsP3-macro	16.28	14.47	29.91	25.55	25.74	13.73	19.42	53.12		58.75	56.25	19.74	21.71	22.37	28.08	30.14
CHIKV-nsP3-macro	13.95	15.79	29.06	21.90	23.53	16.99	17.99	56.88	58.75		89.38	17.76	22.37	22.37	27.40	28.08
ONNV-nsP3-macro	13.18	18.42	29.06	23.36	22.79	16.99	18.71	55.62	56.25	89.38		16.45	21.71	21.05	28.77	28.08
macroH2A1.1	14.86	15.27	17.38	15.09	15.92	22.22	20.24	17.11	19.74	17.76	16.45		18.75	28.42	21.33	24.44
AF1521	15.44	16.09	15.83	23.75	24.05	17.82	27.33	21.71	21.71	22.37	21.71	18.75		29.05	29.41	31.21
ARTD8-macro1	15.89	10.00	20.42	27.04	22.78	24.72	23.64	19.74	22.37	22.37	21.05	28.42	29.05		29.05	28.57
MacroD1	11.27	13.40	21.19	24.84	21.57	19.21	23.90	30.14	28.08	27.40	28.77	21.33	29.41	29.05		53.33
MacroD2	11.72	16.54	16.49	27.10	26.62	20.34	25.16	33.56	30.14	28.08	28.08	24.44	31.21	28.57	53.33	

10-20% | 20-30% | 30-50% | 50-80% | >80%

b

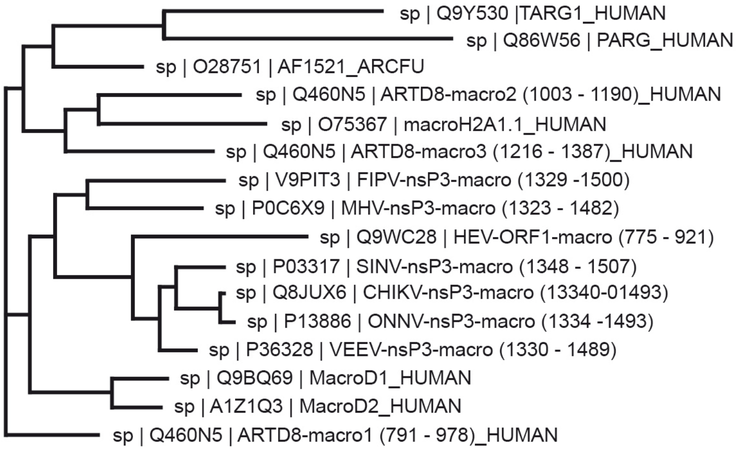

Supplementary Figure S3

(**a**) Percent identity matrix generated with Clustal multiple sequence alignment based on sequence comparison of the indicated macrodomains.

(**b**) Phylogenetic tree created by using ClustalW2.

AF, *Archaeoglobus fulgidus*.

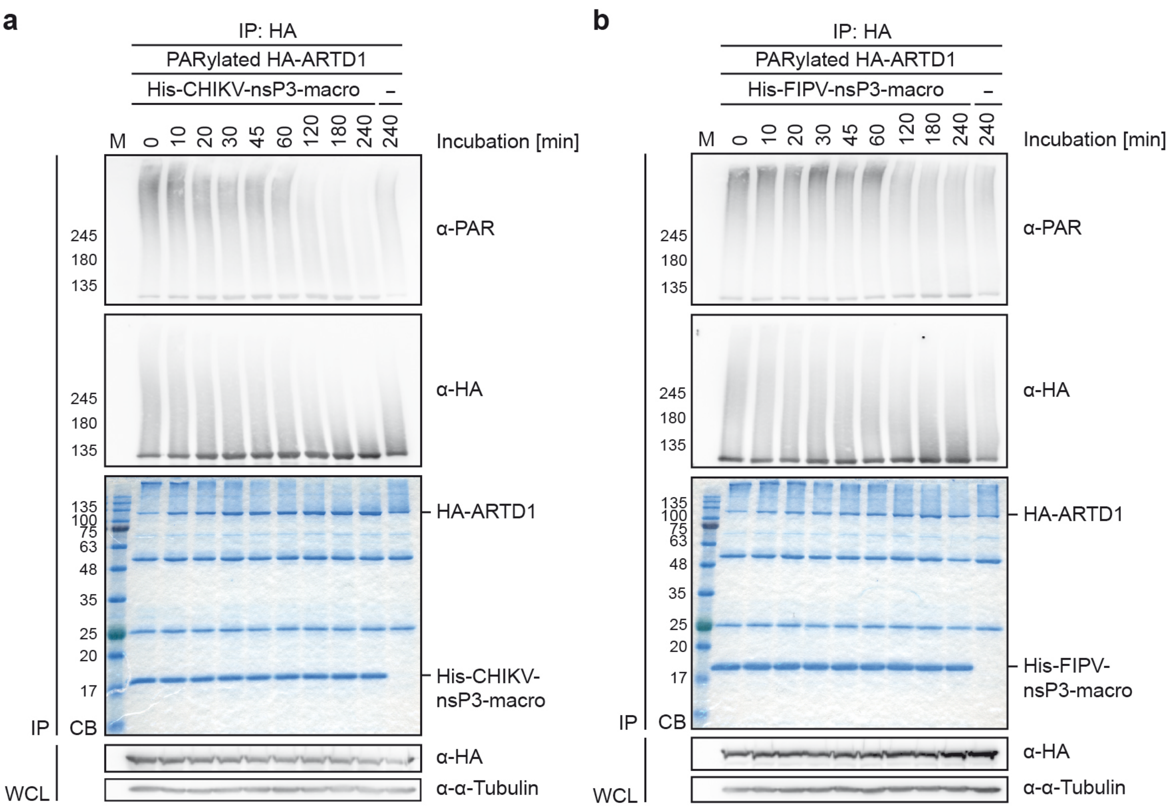

Supplementary Figure S4

(**a**) HA-ARTD1 was expressed in HEK293 cells and immunoprecipitated from lysates. Automodification was carried out in presence of β-NAD^+ and double stranded oligomers. PARylated ARTD1 was then incubated with CHIKV-nsP3 macrodomain for the indicated times. PAR levels were determined using a PAR-specific antibody. Proteins were visualized by Coomassie blue (CB) staining. For control whole cell lysates (WCL) were analyzed for the expression of HA-ARTD1 and α-Tubulin by immunoblotting.

(**b**) As in panel a but with the FIPV-nsP3 macrodomain.

CB, Coomassie blue; IP, immunoprecipitation; WCL, whole cell lysate.

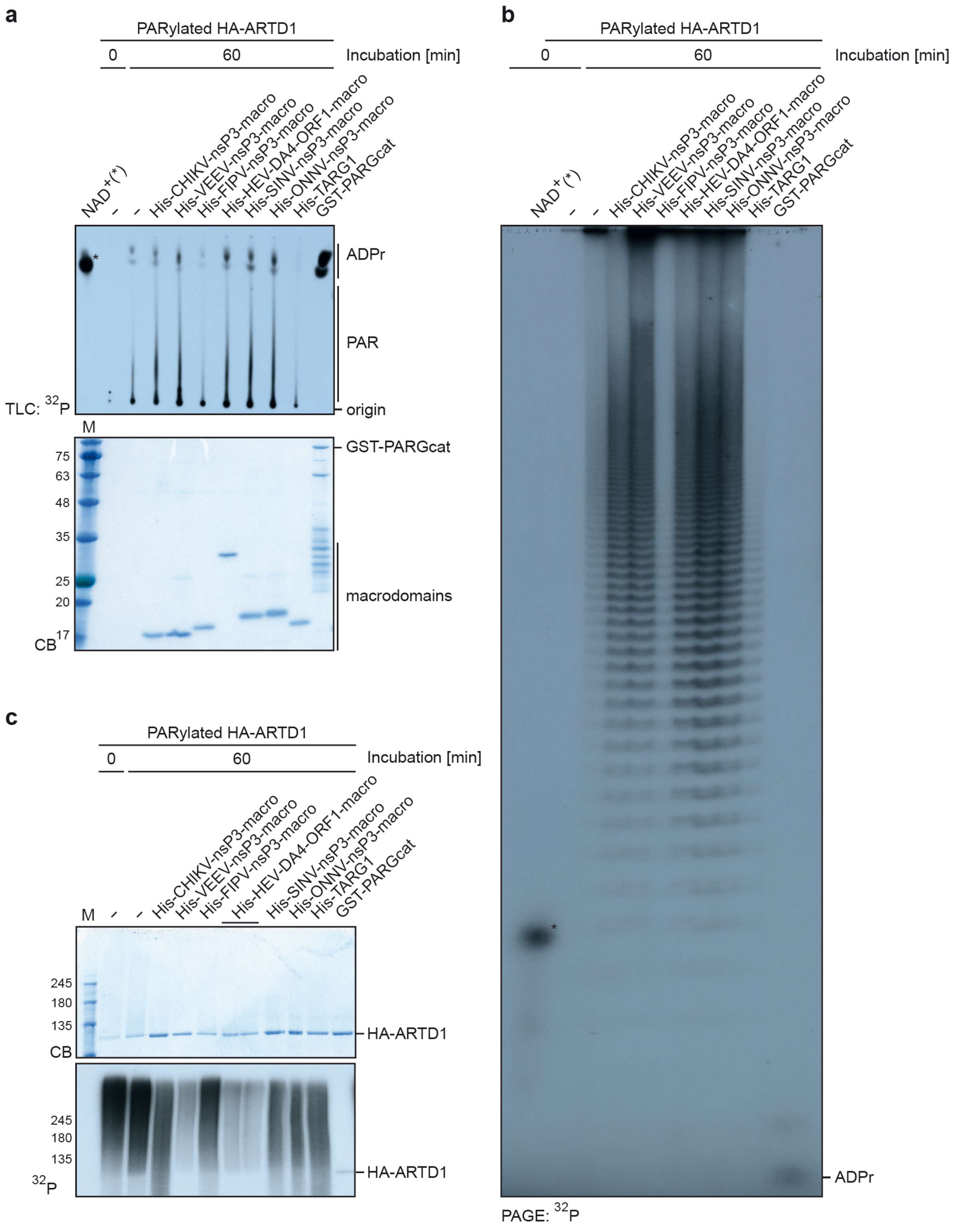
a
PARylated HA-ARTD1
0
60
Incubation [min]
NAD+(*)
-
-
His-CHIKV-nsP3-macro
His-VEEV-nsP3-macro
His-FIPV-nsP3-macro
His-HEV-DA4-ORF1-macro
His-SINV-nsP3-macro
His-ONNV-nsP3-macro
His-TARG1
GST-PARGcat
ADPr
PAR
origin
TLC: 32P
M
75
63
48
35
25
20
17
CB
GST-PARGcat
macrodomains
b
PARylated HA-ARTD1
0
60
Incubation [min]
NAD+(*)
-
-
His-CHIKV-nsP3-macro
His-VEEV-nsP3-macro
His-FIPV-nsP3-macro
His-HEV-DA4-ORF1-macro
His-SINV-nsP3-macro
His-ONNV-nsP3-macro
His-TARG1
GST-PARGcat
ADPr
PAGE: 32P
c
PARylated HA-ARTD1
0
60
Incubation [min]
M
-
-
His-CHIKV-nsP3-macro
His-VEEV-nsP3-macro
His-FIPV-nsP3-macro
His-HEV-DA4-ORF1-macro
His-SINV-nsP3-macro
His-ONNV-nsP3-macro
His-TARG1
GST-PARGcat
245
180
135
CB
HA-ARTD1
245
180
135
32P
HA-ARTD1

Supplementary Figure S5

(**a**) HA-ARTD1 was expressed in HEK293 cells and immunoprecipitated from lysates. Automodification was carried out in presence of ^{32}P-NAD^{+} and double stranded oligomers. PARylated ARTD1 was then subjected to hydrolase assays with the indicated macrodomains. The supernatants were collected and fractions were either analyzed by thin layer chromatography (TLC) to visualize released ADPr and PAR chains or by SDS-PAGE and Coomassie blue (CB) staining to visualize the macrodomains that were used in the hydrolase assays. As control for the TLC analysis ^{32}P-NAD^{+} was spotted onto PEI-F cellulose plates (indicated by *, very left lane).

(**b**) Fractions of the supernatants from panel a were analyzed on a sequencing PAGE to visualize released ADPr and PAR chains. As control ^{32}P-NAD^{+} was used (indicated by *, very left lane).

(**c**) The remaining automodification of immunoprecipitated HA-ARTD1 from panel a (the beads) was analyzed by SDS-PAGE, stained with CB and exposed to X-ray film.

CB, Coomassie blue; SN, supernatant; TLC, thin layer chromatography; ^{32}P, autoradiogram.

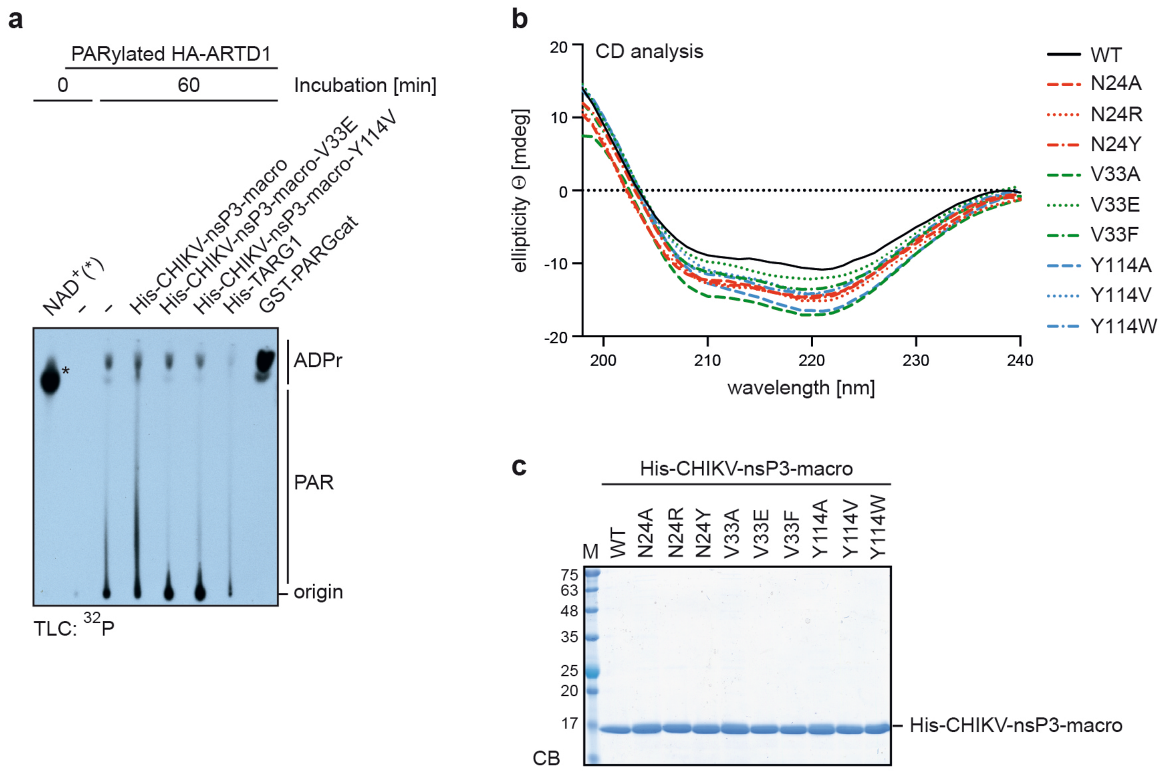

Supplementary Figure S6

(**a**) Transiently expressed HA-ARTD1 was immunoprecipitated from HEK293 cell lysates. Automodification was carried out in presence of ^{32}P-NAD^{+} and double stranded oligomers and ARTD1 was incubated with the CHIKV macrodomain and the indicated mutants. The supernatants were analyzed by thin layer chromatography (TLC) to visualize released ADPr and PAR chains. As control ^{32}P-NAD^{+} was spotted onto PEI-F cellulose plates (indicated by *, very left lane).

(**b**) Circular dichroism (CD) analysis of the CHIKV macrodomain WT and mutants.

(**c**) The different CHIKV macrodomain proteins used for CD analysis in panel a were analyzed by SDS-PAGE and subsequent Coomassie blue staining (CB).

CB, Coomassie blue; CD, circular dichroism; TLC, thin layer chromatography; ^{32}P, autoradiogram.

Supplementary table S1

Calculated Amber score values of the complexes protein/substrate. Bold: WT; blue: primarily tested mutants; red: refined tested mutants.

System	Amber score	System	Amber score	System	Amber score
**WT**	**-5954.0097**	V33N	-6003.613704	Y114R	-6068.869585
N24D	-5927.887207	V33D	-5983.961812	Y114N	-6013.213583
N24G	-5891.917232	V33G	-5955.140954	Y114D	-5990.057064
N24A	-5883.719883	**WT**	**-5954.0097**	Y114Q	-5984.136767
N24C	-5866.822578	V33T	-5952.186938	**WT**	**-5954.0097**
N24P	-5855.335536	V33A	-5950.814242	Y114E	-5949.445958
N24S	-5783.052422	V33C	-5932.209695	Y114M	-5944.486817
N24Q	-5772.444689	V33S	-5929.63734	Y114F	-5937.871572
N24T	-5726.265817	V33P	-5901.034089	Y114S	-5937.599588
N24M	-5323.713256	V33I	-5851.824775	Y114G	-5936.221517
N24E	-5191.599278	V33L	-5630.175383	Y114C	-5922.678038
N24K	-5132.623251	V33H	-5102.607183	Y114H	-5921.85385
N24H	-5010.123918	V33R	-4952.054979	Y114A	-5919.301369
N24L	-4870.223088	V33K	-3332.14082	Y114P	-5864.822403
N24I	-4767.595253	V33M	-1599.684411	Y114L	-5846.35584
N24V	-3940.64876	V33E	-880.050551	Y114K	-5126.708602
N24R	1980.429579	V33Q	392435.7073	Y114T	-4927.906049
N24Y	521107.8312	V33F	496548.1548	Y114V	-993.70063
N24F	552685.4051	V33Y	613383.3739	Y114W	-157.823547
N24W	1285657427	V33W	7926309.849	Y114I	2587047.836
